# Meeting Summary for Keystone Symposia on HIV Cure: Antiretroviral Therapy (ART)-Free Control of HIV Infection in Durban, South Africa, 2025

**DOI:** 10.20411/pai.v10i2.885

**Published:** 2025-10-18

**Authors:** Kiho Tanaka, Tatenda Jimmy Blessing Chikowore, Steven G. Deeks, Jacob D. Estes, Ya-Chi Ho, Sizun Jiang, Ming Jie Lee, Chang Li, Albert Machinda, Mauricio Martins, Patrick Mdletshe, Zaza M. Ndhlovu, Ujjwal Neogi, Melanie M. Ott, Thomas A. Rasmussen, Kavidha Reddy, Rachel L. Rutishauser, Anna Farrell-Sherman, Caroline T. Tiemessen, James E. Voss, Cissy Kityo, Sharon R. Lewin, Thumbi Ndung’u, Joseph M. McCune

**Affiliations:** 1 Department of Infectious Diseases, The University of Melbourne at the Peter Doherty Institute for Infection and Immunity, Melbourne, Australia; 2 Africa Health Research Institute, Durban, South Africa; 3 University College London, London, United Kingdom; 4 Department of Medicine, University of California, San Francisco, San Francisco, California; 5 Vaccine & Gene Therapy Institute, Oregon Health and Science University, Beaverton, Oregon; 6 Department of Microbial Pathogenesis, Yale University School of Medicine, New Haven, Connecticut; 7 Center for Virology and Vaccine Research, Beth Israel Deaconess Medical Center, Harvard Medical School, Boston, Massachusetts; 8 Department of Infectious Disease, Imperial College London, London, United Kingdom; 9 University of Washington, Department of Medicine, Division of Medical Genetics, Seattle, Washington; 10 The Africa HIV Cure Consortium (AHCC) and CIDRZ, Zambia; 11 Department of Immunology and Microbiology, The Herbert Wertheim UF Scripps Institute for Biomedical Innovation & Technology, Jupiter, Florida; 12 Centre for the AIDS Programme of Research in South Africa (CAPRISA), Durban, South Africa; 13 School of Laboratory Medicine and Medical Sciences, College of Health Sciences, University of KwaZulu-Natal, Durban, South Africa; 14 Ragon Institute of Massachusetts General Hospital, Massachusetts Institute of Technology, and Harvard University, Cambridge, Massachusetts; 15 Division of Clinical Microbiology, Department of Laboratory Medicine, Karolinska Institutet, Huddinge, Sweden; 16 Gladstone Institute of Virology, University of California, San Francisco, San Francisco, California; 17 Department of Medicine, University of California, San Francisco, San Francisco, California; 18 Department of Infectious Diseases, Aarhus University Hospital, Aarhus, Denmark; 19 Fred Hutchinson Cancer Center, Vaccine and Infectious Disease Division, Seattle, Washington; 20 Molecular and Cellular Biology PhD Program, University of Washington, Seattle, Washington; 21 Centre for HIV and STIs, National Institutes of Communicable Diseases, a division of the National Health Laboratory Service, and Faculty of Health Sciences, University of the Witwatersrand, Johannesburg, South Africa; 22 Department of Immunology and Microbiology, The Scripps Research Institute, La Jolla, California; 23 Joint Clinical Research Centre (JCRC), Kampala, Uganda; 24 Victorian Infectious Diseases Service, Royal Melbourne Hospital at the Peter Doherty Institute for Infection and Immunity, Melbourne, Australia; 25 Department of Infectious Diseases, Alfred Hospital and Monash University, Melbourne, Australia; 26 HIV Pathogenesis Programme, The Doris Duke Medical Research Institute, University of KwaZulu-Natal, Durban, South Africa; 27 HIV Frontiers, Global Health Accelerator, Gates Foundation, Seattle, Washington

**Keywords:** HIV, Cure, Antiretroviral Therapy-free Control, Reservoir, Persistence, Molecular, Proteomics, Imaging, Multi-omics, Artificial Intelligence, Therapeutics, Broadly Neutralizing Antibodies, Immune Cells, Antiretrovirals, Latency Reversal, Gene Therapy, Animal Models, Clinical Trials, Africa, LMIC, Community

## Abstract

Antiretroviral therapy (ART) can effectively control human immunodeficiency virus (HIV) replication; however, lifelong treatment is required due to viral reservoirs, which fuel viral rebound. This necessitates curative interventions that can achieve either eradication of the reservoir or durable remission off ART. Advances in technology have fostered development of multi-omic techniques encompassing molecular tools, proteomic analyses, imaging, and artificial intelligence (AI)-driven data analysis to understand HIV reservoir biology and persistence. These have informed the investigation of therapeutic interventions such as broadly neutralizing antibodies, latency reversal, immune cell augmentation, antivirals, and gene therapy. From April 7–10, 2025, experts in the field convened at the Keystone Symposia conference, HIV Cure: Antiretroviral Therapy (ART)-Free Control of HIV Infection in Durban, South Africa, to discuss novel strategies for eradication and/or durable ART-free control of HIV.

## INTRODUCTION

Human immunodeficiency virus (HIV) persists despite antiretroviral therapy (ART) due to long-lived reservoirs of latently infected cells, the source of viral rebound [1–3]. People living with HIV (PLWH) must adhere to a lifelong regimen of ART that can prevent disease progression. Nonetheless, PLWH on ART have heightened risks of health complications (such as osteoporosis and cardiovascular disease), face a significant pill burden, and often experience stigma and discrimination [4], each affecting their quality of life and access to care. In resource-limited settings such as low-middle-income countries (LMICs) where the prevalence remains high, universal access to ART has been challenging [5, 6]. Thus, an intervention that leads to durable ART-free control of HIV (aka an “HIV cure”) would have high impact. The Keystone Symposia on HIV Cure: Antiretroviral Therapy (ART)-Free Control of HIV Infection was held in April 2025 in Durban, South Africa, to discuss strategies for eradication or durable ART-free control of HIV, with emphasis on the design and implementation of strategies for LMICs. Current data on biology of the replication competent reservoir and on novel therapeutic approaches were shared among investigators across the world.

## BIOLOGY OF THE REBOUND COMPETENT RESERVOIR

As a definitive property of a retrovirus, HIV integrates a DNA copy of its genome into the host cell genome [7, 8]. ART can effectively inhibit viral replication but is not curative, as clearance of all HIV-harboring cells does not result [9]. Although there is no consensus on a definition of the proviral reservoir [10, 11], it can be described as comprising all cells with integrated HIV proviral DNA genomes in the host nuclear genome [12]. These genomes can then be further characterized as transcriptionally active or silent, and as intact or defective, based on the cellular state and proviral genome integrity. A small proportion of these cells can persist in multiple states and sites harboring replication-competent proviral genomes, forming the “rebound competent reservoir” [8, 12–14]. While earlier concepts of the biology of the HIV reservoir viewed it as a stable snapshot of the viral archive [14], more recent studies have shown it to reflect a dynamic interplay between decay and persistence [15], with early treatment resulting in a reduction in its size and complexity [16, 17]. There is, however, little understanding of the factors that govern the persistence of such cells and the triggers that facilitate viral rebound from them.

### Distribution of the Viral Reservoir

HIV-1 preferentially infects short-lived activated CD4+ T cells; however, studies have shown infection of other cell types [1, 18–20]. A relatively small proportion of these infected cells may persist indefinitely with slow decay rates and a half-life of approximately 44.5 months [21–23]. Early studies of the reservoir characterized its composition based on quiescent long-lived resting memory CD4+ T cells in peripheral blood [8]. Over the last few decades, several technological advances to assess proviral persistence have elucidated the cellular and anatomical distribution of the reservoir, highlighting unique features in distinct compartments of the hematolymphoid system.

### HIV-1 reservoir characteristics in acutely treated adults and children: Implications for HIV cure strategies, Kavidha Reddy

Recent studies have challenged the notion of the restriction of the HIV reservoir to CD4+ T cells, showing productive infection of non-lymphoid cells, including monocytes and several classes of macrophages [12, 20, 24–29]. The HIV reservoir size, landscape, and decay kinetics in lymphocyte and myeloid cellular compartments were assessed in early-treated women from the Females Rising through Education, Support and Health (FRESH) cohort in Durban, South Africa, who were identified during hyperacute infection through twice-weekly HIV RNA testing while HIV negative [30]. Total, intact, and defective HIV DNA measurements from detection up to 1-year post-treatment in peripheral blood mononuclear cells (PBMCs) and sorted memory CD4+ T cell and myeloid subsets were performed with additional viral genome sequence analysis in total PBMCs. The study showed that HIV DNA was detectable soon after infection, confirming the notion of rapid reservoir seeding. The study also highlighted the fact that central and effector memory T cells were the primary reservoir targets and harbored the highest proportion of total HIV DNA, with intact genomes being enriched in effector memory cells. Total HIV DNA was also detectable in myeloid cells, suggesting that reservoir seeding was not limited to CD4+ memory T cells. The impact of ART on the reservoir was assessed and showed that early ART restricted the reservoir largely to CD4+ T cells, whereas delaying ART initiation into chronic infection allowed for the establishment of HIV reservoirs in myeloid cells. Early treatment also limited viral diversity and accelerated decay of intact genomes and could play a key role in strategies aimed at reservoir clearance.

Studies, including the above, have focused on adults and highlight the impact of early ART on the reservoir and disease progression. Children living with HIV, an understudied demographic, may have distinct features as they can initiate ART from birth and have unique immune profiles, which may influence reservoir seeding and persistence. In a study of African children with perinatally acquired HIV who initiated ART ≤90 days (median=34 days) after birth (Early Anti-Retroviral Treatment in Children - EARTH cohort), the total reservoir in peripheral blood was assessed over 4 years to describe factors associated with reservoir size dynamics. Here, the study also showed significant reservoir decay due to early treatment with pre-ART viral load and initial reservoir decay rates predicting the total reservoir size after 3 years. Notably, a smaller reservoir was observed in females, underscoring the need to understand if and how sex differences play a role in the mechanisms of HIV reservoir control. The study also identified children with sustained, virological and immunological control, characterized by undetectable viral load and high CD4+ T cell counts and undetectable HIV-1 DNA reservoir. These children may have a greater likelihood of ART-free viral control due to undetectable circulating reservoir-harboring cells and limited immune disruption and could be good candidates for future analytical treatment interruption trials directed at HIV cure.

### Mechanisms of HIV persistence in tissues - single cell multi-omic and spatial transcriptomic approaches, Ya-Chi Ho

The mechanisms that underlie HIV persistence in tissues are distinct from those found in the peripheral blood [18, 31], with tissue-resident memory T cells (TRMs) providing informative insights into tissue microenvironments and their impact on the differentiation, effector function, migration, proliferation, and survival of HIV proviral reservoir-harboring cells. To assess HIV persistence in tissues, gut-derived CD4+ and CD8+ T cells in 10 PLWH (suppressed on antiretroviral therapy) and 5 people living without HIV (PLWoH) were analyzed to capture viral, transcriptional and cellular profiles using DOGMA-seq [32, 33] and TREK-seq [34, 35]. The results showed higher BACH2 in long-lived TRM CD4+ and CD8+ T cells suggesting that BACH2 drives long-lived memory phenotype in T cells. HIV-specific CD8+ T cell proliferation was greater in response to interferon type 1 (IFN-1) than BACH2, and HIV infection was also more prominent in TRMs in the B-cell follicle, which lacks IFN-1 and CD8+ T cells, creating a tissue microenvironment that may promote HIV persistence. The study found that BACH2 is the leading transcription factor that shapes gut TRMs into long-lived memory in CD4+ TRMs and that restrains interferon-driven effector functions in CD8+ T cells. Although HIV integration into BACH2 promotes the proliferation of HIV-infected cells [36, 37] and represses transcription [38, 39], this study demonstrated that effects of the high BACH2 transcription factor activity in TRMs promotes HIV persistence independent of HIV proviral integration sites. This creates a potential target for interventions aimed at addressing viral persistence.

### Molecular drivers of HIV-persistence and CD8+ T cell dysfunction in human lymphoid tissues during ART-suppressed subtype C infection, Zaza M. Ndhlovu

Several studies have demonstrated the HIV persistence in the lymph nodes (LNs) in PLWH while they are on suppressive ART [31, 40–42]. A cutting-edge study utilizing spatial transcriptomics and high-plex imaging examined the localization of HIV infected cells and CD8+ T cell functionality within the LN tissue microenvironment—germinal centers (GCs) and B cell follicles (BCFs). Here, 10 individuals with suppressed viral loads, 5 with ongoing viremia, and 3 individuals living without HIV were assessed. The study showed that most HIV-infected cells in LNs were germinal center T follicular helper (GC-Tfh) cells localized within BCFs, and approximately 8% of these cells were CD68+ macrophages. These findings were corroborated by the presence of proviral DNA in both CD4+ T cells and macrophages. Interestingly, although the majority of CD8+ T cells express CXCR5 and thus have the potential to enter GCs, they resided outside the follicles. HIV reservoir-harboring (p24+) cells in LNs appeared to be resistant to immune pressure, with adjacent CD8+ T cells showing lower granzyme B production and a unique pattern of epigenetic regulation of granzyme B and perforin genes in comparison to CD8+ T cells in the peripheral blood. A distinct NKG2A+ subset expressing low granzyme B levels was identified within the follicles of PLWH regardless of HIV suppression status, suggesting a localized, yet functionally constrained response. A spatial transcriptomics analysis using the GeoMax platform in areas with HIV+ cells also showed that HIV infection was associated with transcriptional changes in the immune microenvironment, particularly in genes involved in immune regulation. Using these innovative approaches, HIV cure research can be advanced through identification and characterization of non-circulating reservoir sanctuaries and inform the targeting of their clearance.

## MEASURING AND CHARACTERIZING THE RESERVOIR

The HIV reservoir remains a primary barrier to curative efforts and a key feature of HIV persistence [9]. This necessitates accurate and precise tools for its measurement so that strategies aimed at the development of a functional cure or complete viral eradication can be evaluated. The quantitative viral outgrowth assay (QVOA) was the first method used to measure replication-competent and inducible HIV reservoirs in resting CD4+ T cells [1, 3, 43]. Although it is still considered the gold standard, it has several drawbacks, including expense, its time-consumption, the need for large cell numbers, and its underestimation of total reservoir size, as only 1% of latent cells are reactivated under conditions of the protocol [23, 44]. Over the last few decades, several other methods have been developed to address these challenges, including cutting-edge culture protocols, sequencing, polymerase chain reaction (PCR), spatial omics, and modelling and artificial intelligence-based tools, all of which are pushing the boundaries and facilitating a deeper understanding of viral persistence.

### Molecular Approaches to Reservoir Characterization

Advances in PCR techniques have propelled the field, allowing for more rapid and accurate measurements of HIV DNA and/or RNA as a proxy for reservoir size. PCR-based assays can be designed to measure several forms of integrated and extrachromosomal HIV DNA, viral transcripts, and host-viral junctions as integration sites.

### Sequencing-based approach to HIV DNA assessment

Traditional approaches to characterize proviral genomes were based on single-genome sequencing (SGS) of genomic fragments of HIV DNA using the Sanger sequencing platform. This approach provided insight into the diversity of proviruses but was limited in utility due to failure to capture viruses with large deletions and mutations in primer binding sites. Additionally, the assay overestimated the size of the potentially replication-competent reservoir [23, 45]. To address these limitations, the near Full Length Individual Proviral sequencing assay (FLIP-Seq) was developed [46–48]. This assay employs limiting dilution single genome amplification (SGA) of the near full length HIV genome using a nested PCR with subsequent next-generation sequencing (NGS), allowing for a more robust distinction of intact and defective genomes [45]. A companion assay, the Matched Integration site and Proviral Sequencing (MIP-Seq) assay, targets both the provirus and the host-viral junction and has been developed to further describe the chromatin environment of potentially replication-competent genomes [49–51], an environment that could influence the transcriptional activity of such genomes.

### PCR-based detection of HIV DNA

Proviral genome sequencing provides key insights into HIV reservoir characteristics and enables high resolution description of persisting proviruses and their chromatin environment. However, these methods are limited by cost and high sample requirements [23, 45]. In the intact proviral DNA assay (IPDA), estimates of the HIV reservoir size and composition have been widely explored by quantitative amplification of conserved regions of the HIV genome, eg, LTR-*gag* in total HIV DNA, and *psi* and *env* [17, 21, 52, 53]. Advanced platforms using droplet digital PCR have further increased the efficiency and accuracy of these measurements by enabling direct quantification of the target regions. Several studies highlighted here have employed these techniques singly or in multi-omic approaches to assess proviral characteristics, dynamics under viral suppression, and sources of viral rebound post ART interruption.

### Spatial and Multi-omic Tools in Understanding Reservoir Persistence

Tools such as spatial transcriptomics allow for the identification of gene expression by measuring mRNA transcripts at a cellular and/or subcellular level. Spatial profiling captures cytokine gradients, distances between HIV-infected cells and immune effectors, aspects of their functional status, and cell-cell interactions [24]. These tools have been used to study HIV-infected cells in intact tissue sections, assessing changes in tissue microenvironments and their impact on HIV persistence with unprecedented resolution. By integrating the above tools into multi-omic studies, which can further be enhanced using AI foundation models to analyze the multidimensional data, the identification of biomarkers that can predict viral rebound can be accelerated. This will also add to the safety of studies that test interventions aimed at achieving ART-free control of HIV infection, as it would allow for early detection of viral recrudescence.

### Decoding tissue-specific dynamics of SIV rebound in nonhuman primates, Jacob D. Estes

To better understand the kinetics and spatial localization of rebounding viruses, rhesus macaques (RMs) were infected with the barcoded SIVmac239M virus and, after 68 to 70 weeks of suppression on ART, barcode-specific viral RNA expression in tissues was assessed early (5 and 7 days) after treatment interruption. The study employed extensive necropsy tissue sampling from animals on ART, quantitative tracking of vRNA and vDNA matched barcodes in individual tissues (n=413) that established a 99% confidence prediction interval for the spectrum of viral activation during ART. To further understand initial tissue site sources of rebound, multi-omic spatial *in situ* tools were used to quantify the distribution, frequency, clustering, and relative viral RNA expression levels of SIV RNA+ cells in tissue. Molecular barcode analysis was able to precisely identify tissues that reflect rebound-origin sites in most animals with low viremia (1-30 SIV RNA copies/mL) after ART discontinuation and to show that these rebound-origin tissues were enriched in gastrointestinal tract-draining lymph nodes (GDLNs). To understand the early host responses to viral rebound, laser capture tissue proteomics on GDLNs from animals necropsied on ART compared to off ART was performed, showing significant differential protein expression patterns (851 up-regulated and 74 down-regulated) in rebound-origin GDLNs compared to matched GDLNs on ART. Top pathways upregulated in rebound-origin GDLNs included those associated with innate immune system, IFN signaling, antiviral interferon stimulated genes, cytokine signaling, RNA metabolism, and host proteins associated with response to HIV infection. An analysis of GDLNs from animals that were off ART but aviremic (<1 SIV RNA copy/mL) revealed many overlapping differentially expressed proteins/pathways, but to a lesser extent than rebound-origin GDLNs, suggesting host responses may be a sensitive measure of tissue viral rebound that is below the limit of detection of molecular or spatial viral methods. These results indicate that distinct tissue sites differentially restrict/promote post-ART viral rebound and that host responses may be a sensitive measure of viral rebound prior to detecting virus in the plasma. The study highlighted utility of novel multi-omic approaches using nonhuman primate (NHP) models in biomarker discovery and showed how immune responses may provide an early signal of viral rebound, which could inform clinical trial design.

### Technological advancements for high-dimensional spatial analysis of tissue viral rebound using a NHP SIV model, Sizun Jiang

The recently developed PANINI technology [54] represents another methodological break-through in the field, allowing for the simultaneous detection of SIV/HIV viral DNA, RNA, and protein in conjunction with high-dimensional spatial proteomics platforms such as CODEX [55]. The integration of these multiple data modalities provides unprecedented resolution of viral and host interactions within tissue microenvironments. This technology has been applied in several ongoing NHP studies using the chronically SIV-infected RM model. Here, RMs were studied under suppressive ART followed by analytical treatment interruption (ATI), allowing for spatiotemporal analysis of the tissue microenvironment response to ART and then to the impact of viral rebound. Although the complex nature of the generated datasets presents a significant computational challenge, AI foundation models can help identify signature patterns predictive of post-ART viral kinetics, including ones designed specifically for spatial proteomics data [56]. Preliminary results have also suggested that phenotype, function, structure, and specific spatial arrangements of immune cells relative to virally infected cells may predict rebound trajectories with greater accuracy than conventional metrics. Unique clusters of these cells were differentially enriched during ART vs during different phases of ATI. These foundation models may be key in rapidly scaling our understanding of therapeutic strategies that target HIV tissue reservoir clearance and also facilitate the identification of candidate biomarkers predictive of viral rebound. This may be invaluable in reducing dimensionality of multi-omic data and allow for selection of a panel of a combination of molecular, transcriptomic, proteomic, and metabolic markers that can be integrated into studies requiring close monitoring of the effect of interventions.

### Circulating non-viral biomarkers of the rebound competent reservoir, Anna Farrell-Sherman

The identification of circulating biomarkers of viral rebound after interruption of ART would help to define kinetics and mechanisms of rebound and thus be of critical importance in assessing the effectiveness of HIV cure strategies [14, 57]. An intensive longitudinal study was established to explore HIV rebound after treatment interruption in 20 PLWH; 7 were viral controllers (VCs) before initiation of ART. PBMCs and plasma were collected from participants at baseline and then 3 times per week from the time of treatment interruption through treatment reinitiation. Gut and lymph node tissue samples were collected at select timepoints. Multi-omics analyses, including pharmacokinetics, single cell transcriptomics, whole blood transcriptomics, plasma proteomics, plasma metabolomics, characterization of T cells and monocytes by flow cytometry and CyTOF, CD8+ T cell epitope mapping, reservoir measurements, and rebound viral sequencing, were applied to assess potential biomarkers of rebound defined as a VL=200 copies/mL. Samples taken within 7 days before rebound (proximal to rebound) were compared to those taken during the ATI but more than 7 days before rebound (distal to rebound). Differences in monocyte subset composition, plasma metabolomic profiles, and whole blood transcriptomic profiles between distal and proximal timepoints were observed. The study found an increase in CD16++ antiviral monocytes and an upregulation of inflammatory, antiviral, and antigen-presentation genes, including tumor necrosis-factor-α (TNF-α) and IFN-α-associated pathways. These changes in the immune landscape close to the time of rebound present a unique opportunity to develop biomarkers of imminent viral recrudescence with an early window of detection, making them more informative than close viral load monitoring in interventional studies that employ ATI. Additionally, a comparison of circulating plasma molecules showed metabolic markers that distinguished VCs and NCs, suggesting that early immune responses differ between individuals who control viremia compared to non-controllers. There is still ongoing work to validate these and understand the biological pathways underlying these immune responses, but the results have significant implications for development of biomarkers aimed at predicting ART-free viral control.

## THERAPEUTIC APPROACHES FOR CONTROL

An ideal approach towards achieving ART-free virus control would be the development of a “single-shot” intervention that is safe, effective, durable, scalable, affordable, accessible, and acceptable and one that can also prevent reinfection [58]. Multiple approaches have been proposed and investigated, including broadly neutralizing antibodies, enhancing anti-HIV cellular immune responses, compounds to reduce or restrict the replication-competent reservoirs, and gene therapy.

### Broadly Neutralizing Antibodies

Broadly neutralizing antibodies (bNAbs) are antibodies that target conserved regions of the HIV envelope glycoprotein and that can neutralize the virus across multiple clades, making them a potential therapeutic (reviewed in [59]). Many bNAbs have advanced into clinical trials, and combinations of bNAbs have been tested and demonstrated some efficacy in viral control. There remains the need to address issues such as resistance-associated mutations and the half-life of bNAbs (reviewed in [60]).

### The use of long-acting broadly neutralizing antibodies to confer HIV viral control: Results from the RIO study and Gut Sub-study, Ming Jie Lee

The effectiveness of 2 long-acting bNAbs (LS-bNAbs), 10-1074LS [61–64] and 3BNC117LS [65–69], on viral control in the absence of ART was tested in the RIO study [70]. In a 2-stage double-blinded study, 68 participants who started ART during primary or early HIV infection were enrolled and underwent ART interruption after treatment with these 2 bNAbs or normal saline. Results from the first stage comparing bNAbs vs placebo showed, after a single combination dose, the participants receiving the antibodies were 91% less likely to experience viral rebound than those in the placebo group by week 20. Some participants rebounded early (within 20 weeks), some experienced delayed viral rebound, and some (39% of all participants who received LSbNAbs) remained off ART for more than 72 weeks. Of the latter group, 3 participants demonstrated sustained viral suppression after the time when the predicted bNAb concentrations fell below 10 μg/mL. While LS-bNAbs were detected in rectal biopsies up to 24 weeks after a single dose, tissue exposure was nearly 8-fold lower for both bNAbs when compared to blood. Some participants experienced viral rebound prior to 20 weeks after treatment interruption, despite being screened for 10-1074 predicted resistance-associated mutations, highlighting the need for better screening protocols. The participants also experienced viral rebound at serum LS-bNAb concentrations substantially higher than 10 μg/mL, the previously reported efficacy threshold, demonstrating the need for further investigation

### Combination approaches for HIV cure in resource-limited settings, Thumbi Ndung’u

A phase 2a clinical trial of the safety and efficacy of 2 bNAbs (VRC07-523LS [71–73] and CAP256V2LS [74–78]) and a Toll-like receptor-7 agonist (vesatolimod) [79–82] was conducted in 20 women from the FRESH cohort [83] who initiated treatment during the hyperacute phase (NCT05281510). The regimen was found to be generally safe and well-tolerated with no serious adverse events, although 80% of participants experienced mild infusion-related reactions. Three distinct ATI outcomes were observed based on time to meeting ART restart criteria, namely: early restart (<16 weeks), delayed restart (16-44 weeks), and long-term delayed restart or post-treatment control (>44 weeks). Studies are ongoing to understand the mechanisms associated with these outcomes. This African HIV cure trial conducted in women, who are underrepresented in HIV cure research, demonstrates that complex trials can be performed in LMIC settings through multistakeholder collaboration.

### Combination approaches towards eradication or control of HIV, Steven G. Deeks

When ART is interrupted, drug levels fall to subtherapeutic, and local foci of HIV replication then ensue with unique replicating variants rapidly becoming systemic. Then, virus levels increase exponentially, reaching peak viremia within days before eventually diminishing to a host-mediated set-point [84–86] (reviewed in [87]). The post-ART dynamics in those who are destined to control HIV without intervention has been less well-described but likely follows a blunted version of this same pattern [81].

Although there is no single definitive study, the collected experience across several small, experimental studies suggests that bNAbs administered at the right time slow the rate of viral expansion, reduces the level of peak viremia, and eventually leads to lower-setpoints, at least in some; in the interruption studies, the initial slope of the rebound is lower in post-ART controllers than non-controllers, and even lower in the post-bNAb controllers [69, 88–91]. Accordingly, the impact of bNAbs apparently plays out early, when the virus is first beginning to spread.

These observations argue that a strong host-response needs to be present at the time the virus first begins to replicate and spread (the virus:host “intercept”). Emerging data argue that both innate and adaptive immune responses during this period are essential. Indeed, in a small pilot study of SIV neutralizing antibodies administered during an interruption, only those animals with a protective major histocompatibility complex exhibited a response (defined as partial control of SIV at setpoint). In the FRESH [83] study of 20 young women receiving bNABs, having a protective human leukocyte antigen (HLA) allele (class I or II) was strongly associated with a better outcome.

In terms of the HIV cure agenda, these observations, and others, argue that an effective immune response will need to be primed and ready when virus first begins to spread, even before it is detectable systemically. Efforts to induce such responses are underway to target this critical period.

### Elicitation of durable HIV bNAbs from genome-edited B cells as an approach to achieve a functional cure, James E. Voss

Traditional vaccination strategies have failed to elicit bNAb responses due to limitations imposed by the human repertoire of B cell antigen receptors (BCRs) (reviewed in [92]). Taking another approach, a precision genome-editing strategy that expands the repertoire to include bNAb genes in the form of immunoglobulin (Ig) heavy chain-reprogrammed B cells has been developed [93]. These cells express antibody transgenes using cell-endogenous regulatory elements and Ig constant gene exons. This allows for their formatting as functional BCRs that can signal B cell expansion and differentiation or as soluble secreted forms of all isotypes from differentiated plasma cells *in vivo*. In wild-type B6 mice, reprogrammed B6 cells produced boostable VRC01[94–96] responses following immunization with multimeric HIV envelope immunogens, with stable serum levels near effective antiviral concentrations, and the reprogrammed vaccinated repertoire was isotype-switched and somatically mutated. In Indian RMs, VRC01-reprogrammed B cells survived autologous adoptive transfer and generated high serum levels (1 µg/mL). These cells were detected in the periphery, though not as germinal center B cells; serum antibody levels were transient, and the response could not be boosted. Although yet to be clinically translational, this approach is anticipated to combat existing issues with conventional bNAb therapeutics.

### AAV-vectored delivery of HIV bNAbs for tackling pediatric HIV infection, Mauricio Martins

Late diagnoses, limited ART access, and ART adherence remain challenges for children living with HIV-1 (CLWH) [97, 98], underscoring the need for alternative strategies to achieve durable virologic suppression. Although a single administration of adeno-associated virus (AAV) vectors expressing recombinant bNAbs can enable sustained antibody production [99–101] (reviewed in [102]), anti-drug antibodies (ADA) are usually generated, limiting feasibility and preventing re-administration. Reasoning that ADAs may not be generated by the usually tolerant neonatal immune system, the effect of the AAV/bNAb strategy on preventing virus rebound in perinatally simian-HIV (SHIV)-infected infant RMs following ATI was tested. The therapeutic efficacy of AAV-vectored delivery of the HIV-1 bNAb 10-1074 [64] and the immunoadhesin eCD4-Ig [103] was evaluated. In 2 independent studies, AAV vectors were administered alongside ART, initiated at either week 1 (study A) or week 3 (study B) post-infection. All AAV-treated macaques developed stable eCD4-Ig expression, while 10-1074 levels fluctuated due to ADA. Following ATI at week 30, all 6 control monkeys in each study rebounded within 3 weeks. In contrast, of the AAV-treated macaques in studies A and B, ≥80% maintained ART-free control of viremia (*P*=0.0002, 0.0017, respectively), in some cases for >1.5 years. Post-ATI controllers continued to harbor rebound-competent virus, as evidenced by loss of virologic suppression following experimental depletion of CD8+ lymphocytes or AAV-expressed IgG molecules. These findings demonstrate that a single AAV vector administration during early ART initiation can sustain HIV-1 IgG expression at levels sufficient to prevent viral rebound following ATI. If validated in clinical trials, this approach could offer a practical strategy to reduce virologic failure in CLWH.

### Immunological Control of the Virus

Many immune-based strategies that seek to achieve durable viral control aim to elicit an effective HIV-specific CD8+ T cell response. To be effective, these interventions should promote less exhaustible T cell responses targeting antigens less likely to escape (reviewed in [104]). It is not clear which therapeutic interventions augment the function of HIV-specific CD8+ T cells in humans, nor is it known what qualities of HIV-specific CD8+ T cells determine control of the virus after ART is stopped.

### Eliciting effective HIV-specific CD8+ T cell responses, Rachel L. Rutishauser

In a placebo-controlled study of a T cell-based DNA therapeutic vaccine administered to people with HIV on ART (NCT03606213), new HIV-specific T cell responses elicited by the vaccine demonstrated a greater *in vitro* proliferative capacity than did responses that were pre-existing and boosted. A transcriptional signature associated with a robustly boosted clonotype (32x increase in frequency after vaccination) was identified, marked by higher expression of the T cell memory-associated genes *SELL* and *LEF1* as well as an effector marker, *GNLY*, in the cells prior to vaccination.

It is not clear how CD8+ T cell proliferative responses are associated with control of viral rebound after stopping ART. In a proof-of-concept combination immunotherapy clinical trial in 10 people with HIV given a DNA/modified vaccinia Ankara vaccine regimen (targeting conserved elements in Gag; reviewed in [105]) followed by the administration of 2 bNAbs and a Toll-like receptor 9 agonist, followed by the bNAbs given again at the time of an analytical treatment interruption, a larger burst of activated, cycling (Ki-67+) CD8+ T cells with high expression of the T cell memory factor, TCF-1 [106], was found to be associated with lower viral load set points. Taken together, these studies suggest that the efficacy of T cell-based therapeutic strategies may be enhanced by eliciting new T cell responses and promoting a more memory-like differentiation state of HIV-specific CD8+ T cells with high proliferative capacity. Understanding favorable anti-HIV CD8+ T cell responses lays a foundation ground for the design of effective immunotherapeutic approaches.

### Harnessing immunogenetics in the pursuit of pediatric HIV remission, Caroline T. Tiemessen

The HLA region is the most diverse of the human genome, and genetic polymorphisms in HLA genes influence both adaptive and innate immunity (reviewed in [107]). To more broadly assess factors that may restrict HIV-1 reservoir size and HIV control in ART-treated adolescents with perinatally acquired HIV-1 infection, 20 adolescents living with HIV (ALWHs) on ART and 10 adolescents living without HIV (the CHANGES-30 cohort) were enrolled at ~10 years of age. A child from South Africa, born in 2007 with perinatal HIV, who demonstrated post-treatment control and whose viral reservoir and immunological responses were characterised at 9.5 years of age, was used as the reference in this analysis [108]. ALWHs were selected based on the cycle threshold (Ct) values of their viral reservoirs. Those with the largest Ct values (smallest reservoirs) were longitudinally well-controlled on ART, whereas those with among the smallest Ct values (larger reservoirs) were poorly controlled (viral load >1000 RNA copies/mL) and started ART late compared to the well-controlled group. This approach of selecting ALWH from either extreme allowed identification of protective or deleterious factors associated with HIV-1 reservoir size. The 31 ALWHs were HLA genotyped for 11 loci (HLA class I-A -B, and -C and HLA class II-DRB1, -DRB3, -DRB4, -DRB5, -DPB1, -DQB1, -DPA1, and -DQA1) using GenDx NGSgo-MX11-3 kits (sequenced on an Illumina NextSeq platform, analyzed using NGSengine software).

Using the complete HLA profile from 9 of the loci (DRB4 and DRB5 excluded) of the reference South African (SA) child (18 individual HLA alleles), the well-controlled group shared significantly more HLA alleles with the SA case than those poorly controlled (*P*=0.0265). The well-controlled group was enriched (defined as a difference between well- and poorly controlled groups of at least 3 individuals out of 10 possessing a particular allele) for select alleles at 6 loci (5 HLA class II, one HLA class I), while poorly controlled ALWH were enriched for select alleles at 4 HLA loci (3 class II, one class I). No HLA class I alleles known to be associated with HIV control were enriched in either group. Lastly, there was an enrichment of allotypes that engage with NK cells in the well-controlled group, warranting further investigation of the role of NK cells in HIV reservoir control.

### Anti-HIV CAR-T cell therapy, Cissy Kityo

Chimeric antigen receptor (CAR) T cells that are engineered to express surface CD4 and/or bNAbs are being pursued as a strategy to target HIV-infected cells, and multiple CAR-T cell trials are being advanced into clinical trials (reviewed in [109]). A phase 1 trial tested for safety and persistence of CAR T cells that expressed a gp120-specific CAR moiety with siRNA for immune checkpoint molecules [110]. M10 CAR T cells with a CAR molecule to target gp120 (m36.4 to mD1.22) and 10E8scFv-Fc (bNAb) and follicle-homing receptor, CXCR5, were tested in a phase 1 clinical trial [111]. DuoCAR-T cells with 2 different CAR structures with multiple binding sites are also being extensively studied [112–114], and a LVgp120duoCAR-T construct has advanced into a clinical trial to assess safety and its effect on the reservoir (NCT04648046). Preclinical models are assessing bNAb-secreting HIV specific T cells, an innovative approach to strengthen immune responses [115].

One of the challenges of CAR T cells is the complex and costly manufacturing process, posing a barrier to widespread access. There are efforts to develop allogeneic (“off-the-shelf ”) CAR T cell products [116] to address such barriers. Advancements in automated cell engineering and manufacturing protocols and cost-effective bioprocessing technologies can help overcome these hurdles. Recognizing the transformative potential of cell and gene therapies such as CAR T cells for HIV cure, the Global Gene Therapy Initiative (GGTI) was established in 2020 [117]. The initiative aims to drive research collaboration among scientists, clinicians, and policymakers, accelerate regulatory advancements supporting gene therapy approvals, and secure funding strategies to support HIV gene therapy research, especially in regions like sub-Saharan Africa that bear a disproportionate burden of the HIV pandemic.

### *In vivo* HSC gene therapy of HIV/AIDS and SCD with helper-dependent adenovirus vectors, Chang Li

Conventional hematopoietic stem cell (HSC) gene therapies are based on *ex vivo* strategies involving HSC harvesting, culture and modification of the cells in a laboratory, and re-infusion of transduced HSCs following myeloablation. These complex procedures require centralized facilities and are associated with high cost barriers for broad applications. *In vivo* transduction of mobilized HSCs with helper-dependent adenovirus vectors (HDAd) expressing therapeutic transgenes has been developed to treat sickle cell disease, and work is also ongoing to attempt HIV cure [118]. An HDAd vector carrying an optimized HIV decoy receptor, eCD4-Ig [103], was developed and led to sustained serum eCD4-Ig expression after *in vivo* transduction in NHP; serum levels were subtherapeutic for protection against viral challenges with SIVmac239. Precision base editing of CCR5 was performed with HDAd-BE-CCR5KO vectors to knockout CCR5 (reviewed in [119]). R5 HIV-1 infection was inhibited in CD4+ T lymphocytes differentiated *in vitro* from the transduced CD34+ cells. In a humanized mouse model, *in vivo* transduction and selection generated 50% on-target CCR5 editing, which reduced viral load. *In vivo* transduction of NHP cells with HDAd-Combo (a combination vector with both eCD4-Ig gene addition and CCR5 knockout that is equipped with an autoexpansion module) increased base editing at the CCR5 site, indicating a functional *in vivo* selection mechanism. The safety profile and protection against escalating doses of viral challenges will be evaluated in this animal. Its further characterization in animal models will be crucial for the advancement into clinical translation.

### Other Approaches

### Inhibiting the anti-apoptotic factor, BCL-2, as an approach to targeting HIV persistence, Thomas A. Rasmussen

There is increasing evidence that HIV persistence is sustained through intrinsic resistance to cell death of latently infected cells (reviewed in [120]). BCL-2 overexpression has been shown to be a prominent feature in HIV-infected cells that are resistant to killing, and the inducible HIV reservoir has been found to be disproportionately present in BCL-2^hi^ CD4+ T cells [121, 122]. Venetoclax (an inhibitor of BCL-2) has been shown to selectively deplete cells containing intact HIV-DNA in CD4+ T cells from PLWH on ART and to delay the time to viral rebound in HIV-infected humanized mice following discontinuation of ART [123]. Based on these data, the first clinical trial of venetoclax in PLWH on ART (The AMBER Study, EudraCT 2022-001677-31) is currently being conducted in Denmark and Australia. This phase 1/2b dose escalation trial is designed to establish the safety of venetoclax in PLWH on suppressive ART and to test whether inhibition of BCL-2 can sensitize latently infected cells to die. 200mg and 400 mg dose-escalation cohorts are completed. Venetoclax was generally well tolerated. In both dose groups, there was a transient reduction in CD4+ T cell counts. There were no major changes in the composition of memory CD4+ T cell subsets and only minor changes in the expression of activation and exhaustion markers across memory CD4+ T cell subsets. Enrollment into the 600-mg dose group is currently ongoing, with data on the impact of venetoclax on the intact and functional HIV reservoir pending.

### The role of mRNA therapeutics to reverse HIV latency, Sharon R. Lewin

One strategy to eradicate latent HIV is to activate viral transcription, followed by elimination of infected cells through virus-mediated cytotoxicity or immune-mediated clearance; however, current latency-reversing agents have shown limited potency and specificity (reviewed in [120]). To address these issues, an mRNA-lipid nanoparticle (LNP) technology platform was utilized to develop 2 HIV-specific latency reversal agents, including mRNA encoding for Tat or clustered regularly interspaced short palindromic repeats (CRISPR) activation machinery (reviewed in [124, 125]). A new LNP formulation (called LNP-X) was used to efficiently deliver mRNA to resting CD4+ T cells in the absence of cellular toxicity or activation [126]. Compared to the Onpattro LNP formulation (summarised in [127]) that has been licensed for the treatment of a rare form of amyloidosis, LNP-X had higher association with the target cell and more efficient endosomal release of the RNA, enabling expression of mCherry in over 80% of resting cells [126].

LNP-X encapsulating an mRNA encoding exon 1 of the HIV Tat protein increased HIV transcription in latently-infected cell lines and *ex vivo* in CD4+ T cells from PLWH on antiretroviral therapy [126]. In these primary cells, Tat mRNA delivered by LNP-X increased production of cell associated multiply spliced transcripts (~110 fold above dimethyl sulfoxide control) and HIV RNA in supernatant to levels comparable those after stimulation with Phorbol 12-myristate 13-acetate [126]. Surprisingly, there was no decline in intact proviral DNA. LNP-X further enabled the delivery of CRISPR activation machinery together with guide RNAs that bind the HIV LTR and increased cell-associated HIV RNA transcripts above background, though the magnitude of increase was modest [126]. Using CRISPR activation to increase expression of the host gene CD25, only 25% of cells were expressing the host gene, demonstrating that more efficient delivery of CRISPR activation mRNA is needed [126]. Finally, when Tat mRNA was delivered together with CRISPR activation mRNA to a latently-infected cell line, there was a synergistic increase in activation of the LTR [126]. The impact of these next generation HIV-specific mRNA latency-reversing agents *in vivo* is currently under investigation.

### Silencing the transcriptionally active HIV reservoir, Melanie M. Ott

Persistent viral transcription can drive chronic immune activation through multiple mechanisms: engaging innate nucleic acid sensing pathways, generating viral proteins that induce inflammatory responses, and driving expansion of the viral reservoir through antigen-driven clonal expansion of HIV-specific infected T cells (reviewed in [128, 129]). In the concept of “curative ART,” conventional ART is complemented with a transcriptional inhibitor to suppress chronic inflammation and delay viral rebound. The naturally derived compound didehydro-cortistatin A (dCA) is a potent Tat inhibitor [130], disrupting the feedback mechanism of the virally encoded Tat protein on HIV transcription. In both *in vitro* and *in vivo* models, dCA supplementation with ART accelerated viral suppression and inhibited viral rebound after treatment interruption [131]. Other promising Tat inhibitors currently under investigation are therapeutic approaches to induce permanent changes in the viral chromatin structure, including CRISPR-based silencing strategies. While this is still preclinical, complementing current ART regimens with HIV transcriptional inhibitors offers a strategy to silence the viral promoter, potentially reducing viral gene products below inflammatory thresholds, permitting both direct and indirect mechanisms for durable viral control.

### Mitochondrial dysregulation and HIV latency: A metabolic pathway to a functional cure? Ujjwal Neogi

The application of context-specific Genome-Scale Metabolic Models (GEMs) enables an in-depth mapping of metabolic fluxes at bulk and single-cell resolution [132]. These models uncover disease- and cell-specific metabolic alterations, setting the stage for targeted interventions. By applying the metabolic models, recent studies on elite controllers (ECs) and long-term PLWH have revealed a novel immunometabolic target [133][134]. By integrating data from several cohorts (eg, India, Cameroon, Sweden, Denmark, and the Netherlands) and applying multi-omics analysis, immune-phenotyping, and metabolic modeling, a druggable molecular network regulating α-ketoglutarate (AKG) homeostasis was identified. This was disrupted in PLWH on ART and may drive the persistence of latent HIV through metabolic reprogramming in myeloid lineage cells.

Metabolic modeling predicted disrupted AKG homeostasis due to mitochondrial biogenesis in the pre-monocytic latent cell line U1 cells during viral activation, and metabolic measurements confirmed increased glycolytic activity and altered the citric acid (TCA) cycle intermediates. This metabolic reprogramming drove U1 cells towards an M2-like anti-inflammatory macrophage phenotype, resembling the phenotype seen among PLWH. While still exploratory, these findings suggest metabolic events are among the drivers of immune dysfunction in ART-treated PLWH, and novel interventions should also aim to restore the metabolic balance that can aid HIV cure strategies.

**Figure 1.**
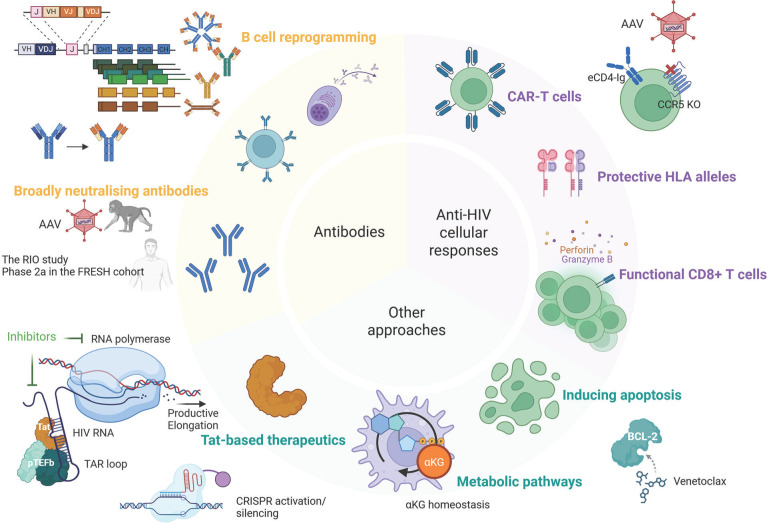
**Therapeutic approaches for ART-free control of HIV**. Many approaches have been investigated to achieve ART-free control of HIV, including those using broadly neutralizing antibodies, strategies to improve anti-HIV cellular responses, and other interventions such as Tat-based therapeutics, targeting metabolic pathways and apoptosis induction by the BCL-2 inhibitor, venetoclax. Of note, many strategies involved gene editing approaches, such as AAV delivery of broadly neutralising monoclonal antibodies, CAR moieties, CCR5 knockdown, and the use of CRISPR to induce or silence transcription of latent HIV and to genetically reprogram B cells.

## UNIVERSAL ACCESS TO CONTROL

### The Africa HIV Cure Consortium: Partnerships for Discovery, Development, and Roll Out of HIV Cure Initiatives in Africa, Albert Machinda

The Africa HIV Cure Consortium (AHCC) is a pioneering Pan-African initiative, which grew out of the public-private partnership known as the HIV Cure Africa Acceleration Partnership (HCAAP) [135] and is focused on ensuring African leadership in the discovery, development, and delivery of an HIV cure. Recognizing Africa’s disproportionate HIV burden and paucity of HIV cure research, the AHCC is built on the principle that people in Africa must not only participate but lead the global HIV cure agenda. The consortium is uniting a diverse group of African stakeholders, framing its work through a Pan-African lens to ensure local ownership, sustainable solutions, and lasting impact. Launched in 2024 with support from the Gates Foundation and Aidsfonds, the AHCC unites global partners to establish a sustainable HIV Cure ecosystem grounded in African leadership, policy integration, and community engagement. With 7 founding organizations, including the Centre for Infectious Disease Research in Zambia (CIDRZ), African Alliance, Sub-Saharan African Network for TB/HIV Research Excellence (SANTHE), AIDS Vaccine Advocacy Coalition (AVAC), International AIDS Society (IAS), the HIV Cure Africa Acceleration Partnership (HCAAP), and the Global Gene Therapy Initiative (GGTI), the consortium brings together expertise from multidisciplinary areas such as clinical research, advocacy, and civil society.

The consortium utilizes a 3-pronged partnerships model: partnerships for discovery, development, and roll-out. In the discovery phase, partners like SANTHE and GGTI are leading African research networks and clinical trials. For development, HCAAP shapes target product profiles for scalable cures, while AVAC strengthens community understanding and advocacy. During roll-out, CIDRZ coordinates policy integration and consortium governance, while partners such as the African Alliance embed cure strategies into national HIV plans. Global allies like IAS and AVAC further amplify African voices within international regulatory and scientific platforms, including the Africa Centres for Disease Control and Prevention (CDC) and the World Health Organization African Region (WHO AFRO). Crucially, the AHCC prioritizes community engagement and ethical trial design, ensuring that cure research reflects local realities, is accepted by affected populations, and is delivered in an ethical manner.

Recent activities highlight AHCC’s momentum: the West and Central Africa HIV Cure Agenda meeting in Cameroon (May 2025), the Research-for-Cure Academy in South Africa (June 2025), piloting a novel HIV Cure Curriculum in Zambia (June 2025), and a high-level Cure Agenda Roundtable in Rwanda (July 2025).

### Community Engagement for HIV Cure, Patrick Mdletshe

Communities play a vital role in shaping and advancing scientific research, particularly in the context of HIV. A future HIV cure carries profound meaning for communities. It is not only about freedom from lifelong treatment or the burden of daily pills, injections, and the risk of drug resistance. A cure would also represent an end to stigma and discrimination, offer hope for relationships and family life, reduce economic strain, and provide healing from the emotional trauma carried by many living with HIV. Most importantly, it would restore dignity and empower individuals to fully participate in society.

Communities must not be viewed as junior partners in research or for compliance. Instead, they should be treated as equal collaborators. Lessons from past failed interventions (eg, the underwhelming uptake of pre-exposure prophylaxis despite its proven effectiveness) underscore what happens when communities are not fully engaged. For acceptance of and equitable access to novel therapeutics, community engagement that respects community priorities is inevitable. A truly effective research process must include community voices from the beginning. Communities are faced with many challenges; a holistic approach is important, prioritizing the mental health and well-being of participants, respecting the role of community advisory boards as meaningful platforms for dialogue and not just formalities, and acknowledging socio-economic realities, including the risks of exploitation due to unemployment. Community members bring more than consent; they also bring commitment, experience, and insight. For research to succeed and for interventions to be accepted, the community must work in true partnership with all other members of the team working to develop a cure for HIV. “Nothing About Us Without Us.”

## CONCLUSION

The 2025 Keystone Symposia on HIV Cure: Antiretroviral Therapy (ART)-Free Control of HIV Infection provided insights into research aimed at exploring the factors that govern the biology of the replication-competent reservoir. Novel therapeutic approaches were discussed, aiming to develop safe and effective curative interventions for HIV that can be delivered acceptably to all in need. Characteristics of the HIV reservoir were explored, evaluating replication competency, cellular activation and metabolic state, location, and the environment in peripheral blood and tissues. Therapeutic strategies, including bNAbs, immune modulation, and gene therapy, were discussed. Hand in hand with innovative strategies in biomarker discovery for evaluation of effectiveness of curative interventions, a keen focus on community-informed research in implementation of these studies was observed.

The symposium facilitated discussion and exchange of ideas as to how we might realistically achieve HIV cure. The meeting also underscored the need for community engagement as a key component of research aimed at cure and the need to address the translational gap in LMIC to assure that curative interventions, if developed, are accessible where most needed. It also stressed the necessity for multistakeholder engagement and leadership in the regions. Biomarkers of viral rebound will help test therapeutic interventions. Critically, a cure must be safe, effective, durable, accessible, affordable, and acceptable and should include strategies to prevent reinfection upon subsequent exposure.

Although ART has greatly improved the quality of life of PLWH, it is not curative and presents socio-economic challenges. Given the current state of ART provision and delivery to areas of the world where HIV disease is prevalent [136], now more than ever, there is a need for curative interventions that can be brought to all in need, enabling better health, preventing transmission to those now uninfected, and ultimately ending the pandemic

## References

[1] Finzi D, Hermankova M, Pierson T, Carruth LM, Buck C, Chaisson RE, Quinn TC, Chadwick K, Margolick J, Brookmeyer R, Gallant J, Markowitz M, Ho DD, Richman DD, Siliciano RF. Identification of a reservoir for HIV-1 in patients on highly active antiretroviral therapy. Science. 1997;278(5341):1295-300. doi: 10.1126/science.278.5341.1295. PubMed PMID: 9360927. 9360927

[2] Chun TW, Stuyver L, Mizell SB, Ehler LA, Mican JA, Baseler M, Lloyd AL, Nowak MA, Fauci AS. Presence of an inducible HIV-1 latent reservoir during highly active antiretroviral therapy. Proc Natl Acad Sci U S A. 1997;94(24):13193-7. doi: 10.1073/pnas.94.24.13193. PubMed PMID: 9371822; PMCID: PMC24285. 9371822 PMC24285

[3] Wong JK, Hezareh M, Gunthard HF, Havlir DV, Ignacio CC, Spina CA, Richman DD. Recovery of replication-competent HIV despite prolonged suppression of plasma viremia. Science. 1997;278(5341):1291-5. doi: 10.1126/science.278.5341.1291. PubMed PMID: 9360926. 9360926

[4] Chawla A, Wang C, Patton C, Murray M, Punekar Y, de Ruiter A, Steinhart C. A Review of Long-Term Toxicity of Antiretroviral Treatment Regimens and Implications for an Aging Population. Infect Dis Ther. 2018;7(2):183-95. doi: 10.1007/s40121-018-0201-6. PubMed PMID: 29761330; PMCID: PMC5986685. 29761330 PMC5986685

[5] Rosenberg NE, Shook-Sa BE, Liu M, Stranix-Chibanda L, Yotebieng M, Sam-Agudu NA, Hudgens MG, Phiri SJ, Mutale W, Bekker LG, Moyo S, Zuma K, Charurat ME, Justman J, Chi BH. Adult HIV-1 incidence across 15 high-burden countries in sub-Saharan Africa from 2015 to 2019: a pooled analysis of nationally representative data. Lancet HIV. 2023;10(3):e175-e85. doi: 10.1016/S2352-3018(22)00328-9. PubMed PMID: 36702151; PMCID: PMC10126805. 36702151 PMC10126805

[6] Babatunde AO, Akin-Ajani OD, Abdullateef RO, Togunwa TO, Isah HO. Review of antiretroviral therapy coverage in 10 highest burden HIV countries in Africa: 2015-2020. J Med Virol. 2023;95(1):e28320. doi: 10.1002/jmv.28320. PubMed PMID: 36397202. 36397202

[7] Craigie R, Bushman FD. HIV DNA integration. Cold Spring Harb Perspect Med. 2012;2(7):a006890. doi: 10.1101/cshperspect.a006890. PubMed PMID: 22762018; PMCID: PMC3385939. 22762018 PMC3385939

[8] Cohn LB, Chomont N, Deeks SG. The Biology of the HIV-1 Latent Reservoir and Implications for Cure Strategies. Cell Host & Microbe. 2020;27(4):519-30. doi: 10.1016/j.chom.2020.03.014. 32272077 PMC7219958

[9] Ndung’u T, McCune JM, Deeks SG. Why and where an HIV cure is needed and how it might be achieved. Nature. 2019;576(7787):397-405. doi: 10.1038/s41586-019-1841-8. 31853080 PMC8052635

[10] Churchill MJ, Deeks SG, Margolis DM, Siliciano RF, Swanstrom R. HIV reservoirs: what, where and how to target them. Nature Reviews Microbiology. 2016;14(1):55-60. doi: 10.1038/nrmicro.2015.5. 26616417

[11] Bruner KM, Hosmane NN, Siliciano RF. Towards an HIV-1 cure: measuring the latent reservoir. Trends Microbiol. 2015;23(4):192-203. doi: 10.1016/j.tim.2015.01.013. PubMed PMID: 25747663; PMCID: PMC4386620. 25747663 PMC4386620

[12] Murzin AI, Elfimov KA, Gashnikova NM. The Proviral Reservoirs of Human Immunodeficiency Virus (HIV) Infection. Pathogens. 2025;14(1):15. PubMed PMID: doi: 10.3390/pathogens14010015. PMC11768375 39860976

[13] Wang Z, Simonetti FR, Siliciano RF, Laird GM. Measuring replication competent HIV-1: advances and challenges in defining the latent reservoir. Retrovirology. 2018;15(1):21. doi: 10.1186/s12977-018-0404-7. 29433524 PMC5810003

[14] Deeks SG, Archin N, Cannon P, Collins S, Jones RB, de Jong MAWP, Lambotte O, Lamplough R, Ndung’u T, Sugarman J, Tiemessen CT, Vandekerckhove L, Lewin SR, International AIDS Society (IAS) Global Scientific Strategy working group. Research priorities for an HIV cure: International AIDS Society Global Scientific Strategy 2021. Nature Medicine. 2021;27(12):2085-98. doi: 10.1038/s41591-021-01590-5. 34848888

[15] Kreider EF, Bar KJ. HIV-1 Reservoir Persistence and Decay: Implications for Cure Strategies. Curr HIV/AIDS Rep. 2022;19(3):194-206. doi: 10.1007/s11904-022-00604-2. PubMed PMID: 35404007; PMCID: PMC10443186. 35404007 PMC10443186

[16] Lee GQ, Reddy K, Einkauf KB, Gounder K, Chevalier JM, Dong KL, Walker BD, Yu XG, Ndung’u T, Lichterfeld M. HIV-1 DNA sequence diversity and evolution during acute subtype C infection. Nature Communications. 2019;10(1):2737. doi: 10.1038/s41467-019-10659-2. PMC6588551 31227699

[17] Reddy K, Lee GQ, Reddy N, Chikowore TJB, Baisley K, Dong KL, Walker BD, Yu XG, Lichterfeld M, Ndung’u T. Differences in HIV-1 reservoir size, landscape characteristics and decay dynamics in acute and chronic treated HIV-1 Clade C infection. medRxiv. 2024:2024.02.16.24302713. doi: 10.1101/2024.02.16.24302713. PMC11841988 39976231

[18] Banga R, Perreau M. The multifaceted nature of HIV tissue reservoirs. Current Opinion in HIV and AIDS. 2024;19(3):116-23. doi: 10.1097/coh.0000000000000851. PubMed PMID: 01222929-202405000-00006. 38547340 PMC10990014

[19] Chun TW, Engel D, Berrey MM, Shea T, Corey L, Fauci AS. Early establishment of a pool of latently infected, resting CD4(+) T cells during primary HIV-1 infection. Proc Natl Acad Sci U S A. 1998;95(15):8869-73. doi: 10.1073/pnas.95.15.8869. PubMed PMID: 9671771; PMCID: PMC21169. 9671771 PMC21169

[20] Alexaki A, Liu Y, Wigdahl B. Cellular reservoirs of HIV-1 and their role in viral persistence. Curr HIV Res. 2008;6(5):388-400. doi: 10.2174/157016208785861195. PubMed PMID: 18855649; PMCID: PMC2683678. 18855649 PMC2683678

[21] McMyn NF, Varriale J, Fray EJ, Zitzmann C, MacLeod H, Lai J, Singhal A, Moskovljevic M, Garcia MA, Lopez BM, Hariharan V, Rhodehouse K, Lynn K, Tebas P, Mounzer K, Montaner LJ, Benko E, Kovacs C, Hoh R, Simonetti FR, Laird GM, Deeks SG, Ribeiro RM, Perelson AS, Siliciano RF, Siliciano JM. The latent reservoir of inducible, infectious HIV-1 does not decrease despite decades of antiretroviral therapy. The Journal of Clinical Investigation. 2023;133(17). doi: 10.1172/JCI171554. PMID: 37463049; PMCID: PMC10471168. PMC10471168 37463049

[22] Pasternak AO, Berkhout B. HIV persistence: silence or resistance? Current Opinion in Virology. 2023;59:101301. doi: 10.1016/j.coviro.2023.101301. 36805974

[23] Falcinelli SD, Ceriani C, Margolis DM, Archin NM. New Frontiers in Measuring and Characterizing the HIV Reservoir. Front Microbiol. 2019;10:2878. doi: 10.3389/fmicb.2019.02878. PubMed PMID: 31921056; PMCID: PMC6930150. 31921056 PMC6930150

[24] Zaman F, Smith ML, Balagopal A, Durand CM, Redd AD, Tobian AAR. Spatial technologies to evaluate the HIV-1 reservoir and its microenvironment in the lymph node. mBio. 2024;15(8):e01909-24. doi: 10.1128/mbio.01909-24. 39058091 PMC11324018

[25] Veenhuis RT, Abreu CM, Shirk EN, Gama L, Clements JE. HIV replication and latency in monocytes and macrophages. Seminars in Immunology. 2021;51:101472. doi: 10.1016/j.smim.2021.101472. 33648815 PMC10171083

[26] Sonza S, Mutimer HP, Oelrichs R, Jardine D, Harvey K, Dunne A, Purcell DF, Birch C, Crowe SM. Monocytes harbour replication-competent, non-latent HIV-1 in patients on highly active antiretroviral therapy. AIDS. 2001;15(1):17-22. PubMed PMID: 00002030-200101050-00005. 11192864 10.1097/00002030-200101050-00005

[27] Xu Y, Zhu H, Wilcox CK, van’t Wout A, Andrus T, Llewellyn N, Stamatatos L, Mullins JI, Corey L, Zhu T. Blood Monocytes Harbor HIV Type 1 Strains with Diversified Phenotypes Including Macrophage-Specific CCR5 Virus. The Journal of Infectious Diseases. 2008;197(2):309-18. doi: 10.1086/524847. 18173363

[28] Wong ME, Jaworowski A, Hearps AC. The HIV Reservoir in Monocytes and Macrophages. Front Immunol. 2019;10:1435. doi: 10.3389/fimmu.2019.01435. PubMed PMID: 31297114; PMCID: PMC6607932. 31297114 PMC6607932

[29] León-Rivera R, Veenstra M, Donoso M, Tell E, Eugenin Eliseo A, Morgello S, Berman Joan W. Central Nervous System (CNS) Viral Seeding by Mature Monocytes and Potential Therapies To Reduce CNS Viral Reservoirs in the cART Era. mBio. 2021;12(2):10.1128/mbio.03633-20. doi: 10.1128/mbio.03633-20. PMC8092320 33727362

[30] Dong KL, Moodley A, Kwon DS, Ghebremichael MS, Dong M, Ismail N, Ndhlovu ZM, Mabuka JM, Muema DM, Pretorius K, Lin N, Walker BD, Ndung’u T. Detection and treatment of Fiebig stage I HIV-1 infection in young at-risk women in South Africa: a prospective cohort study. Lancet HIV. 2018;5(1):e35-e44. doi: 10.1016/s2352-3018(17)30146-7. PubMed PMID: 28978417; PMCID: PMC6506720. 28978417 PMC6506720

[31] Pieren D, Benítez-Martínez A, Genescà M. Targeting HIV persistence in the tissue. Current Opinion in HIV and AIDS. 2023;19. doi: 10.1097/COH.0000000000000836. 38169333

[32] Mimitou EP, Lareau CA, Chen KY, Zorzetto-Fernandes AL, Hao Y, Takeshima Y, Luo W, Huang TS, Yeung BZ, Papalexi E, Thakore PI, Kibayashi T, Wing JB, Hata M, Satija R, Nazor KL, Sakaguchi S, Ludwig LS, Sankaran VG, Regev A, Smibert P. Scalable, multimodal profiling of chromatin accessibility, gene expression and protein levels in single cells. Nature Biotechnology. 2021;39(10):1246-58. doi: 10.1038/s41587-021-00927-2. PMID: 34083792; PMCID: PMC8763625. PMC8763625 34083792

[33] Xu Z, Heidrich-O’Hare E, Chen W, Duerr RH. Comprehensive benchmarking of CITE-seq versus DOGMA-seq single cell multimodal omics. Genome Biology. 2022;23(1):135. doi: 10.1186/s13059-022-02698-8. 35739535 PMC9219143

[34] Tu AA, Gierahn TM, Monian B, Morgan DM, Mehta NK, Ruiter B, Shreffler WG, Shalek AK, Love JC. TCR sequencing paired with massively parallel 3’ RNA-seq reveals clonotypic T cell signatures. Nature Immunology. 2019;20(12):1692-9. doi: 10.1038/s41590-019-0544-5. 31745340 PMC7528220

[35] Miller TE, Lareau CA, Verga JA, DePasquale EAK, Liu V, Ssozi D, Sandor K, Yin Y, Ludwig LS, El Farran CA, Morgan DM, Satpathy AT, Griffin GK, Lane AA, Love JC, Bernstein BE, Sankaran VG, van Galen P. Mitochondrial variant enrichment from high-throughput single-cell RNA sequencing resolves clonal populations. Nature Biotechnology. 2022;40(7):1030-4. doi: 10.1038/s41587-022-01210-8. PMC9288977 35210612

[36] Wagner TA, McLaughlin S, Garg K, Cheung CY, Larsen BB, Styrchak S, Huang HC, Edlefsen PT, Mullins JI, Frenkel LM. HIV latency. Proliferation of cells with HIV integrated into cancer genes contributes to persistent infection. Science. 2014;345(6196):570-3. doi: 10.1126/science.1256304. PubMed PMID: 25011556; PMCID: PMC4230336. 25011556 PMC4230336

[37] Maldarelli F, Wu X, Su L, Simonetti FR, Shao W, Hill S, Spindler J, Ferris AL, Mellors JW, Kearney MF, Coffin JM, Hughes SH. HIV latency. Specific HIV integration sites are linked to clonal expansion and persistence of infected cells. Science. 2014;345(6193):179-83. doi: 10.1126/science.1254194. PubMed PMID: 24968937; PMCID: PMC4262401. 24968937 PMC4262401

[38] Yao C, Lou G, Sun H-W, Zhu Z, Sun Y, Chen Z, Chauss D, Moseman EA, Cheng J, D’Antonio MA, Shi W, Shi J, Kometani K, Kurosaki T, Wherry EJ, Afzali B, Gattinoni L, Zhu Y, McGavern DB, …, Wu T. BACH2 enforces the transcriptional and epigenetic programs of stem-like CD8+ T cells. Nature Immunology. 2021;22(3):370-80. doi: 10.1038/s41590-021-00868-7. 33574619 PMC7906956

[39] Igarashi K, Kurosaki T, Roychoudhuri R. BACH transcription factors in innate and adaptive immunity. Nat Rev Immunol. 2017;17(7):437-50. doi: 10.1038/nri.2017.26. PubMed PMID: 28461702. 28461702

[40] Mahlobo B, Laher F, Smidt W, Ogunshola F, Khaba T, Nkosi T, Mbatha A, Ngubane T, Dong K, Jajbhay I, Pansegrouw J, Ndhlovu ZM. The impact of HIV infection on the frequencies, function, spatial localization and heterogeneity of T follicular regulatory cells (TFRs) within human lymph nodes. BMC Immunology. 2022;23(1):34. doi: 10.1186/s12865-022-00508-1. 35778692 PMC9250173

[41] Baiyegunhi OO, Mann J, Khaba T, Nkosi T, Mbatha A, Ogunshola F, Chasara C, Ismail N, Ngubane T, Jajbhay I, Pansegrouw J, Dong KL, Walker BD, Ndung’u T, Ndhlovu ZM. CD8 lymphocytes mitigate HIV-1 persistence in lymph node follicular helper T cells during hyperacute-treated infection. Nature Communications. 2022;13(1):4041. doi: 10.1038/s41467-022-31692-8. PMC9279299 35831418

[42] Li K, Liu B, Ma R, Zhang Q. HIV Tissue Reservoirs: Current Advances in Research. AIDS Patient Care and STDs. 2023;37(6):284-96. doi: 10.1089/apc.2023.0028. PubMed PMID: 37184898. 37184898

[43] Siliciano JD, Siliciano RF. Enhanced culture assay for detection and quantitation of latently infected, resting CD4+ T-cells carrying replication-competent virus in HIV-1-infected individuals. Methods Mol Biol. 2005;304:3-15. doi: 10.1385/1-59259-907-9:003. PMID: 16061962. 16061962

[44] Zhang X, Chen J. HIV Reservoir: How to Measure It? Current HIV/AIDS Reports. 2023;20(2):29-41. doi: 10.1007/s11904-023-00653-1. 37004676

[45] Moar P, Premeaux Thomas A, Atkins A, Ndhlovu Lishomwa C. The latent HIV reservoir: current advances in genetic sequencing approaches. mBio. 2023;14(5):e01344-23. doi: 10.1128/mbio.01344-23. 37811964 PMC10653892

[46] Lee GQ, Orlova-Fink N, Einkauf K, Chowdhury FZ, Sun X, Harrington S, Kuo H-H, Hua S, Chen H-R, Ouyang Z, Reddy K, Dong K, Ndung’u T, Walker BD, Rosenberg ES, Yu XG, Lichterfeld M. Clonal expansion of genome-intact HIV-1 in functionally polarized Th1 CD4+ T cells. The Journal of Clinical Investigation. 2017;127(7):2689-96. doi: 10.1172/JCI93289. 28628034 PMC5490740

[47] Lee GQ, Lichterfeld M. Near-Full-Length Single-Genome HIV-1 DNA Sequencing. Methods Mol Biol. 2022;2407:357-64. doi: 10.1007/978-1-0716-1871-4_23. PubMed PMID: 34985675; PMCID: PMC9135474. 34985675 PMC9135474

[48] Wang XQ, Palmer S. Single-molecule techniques to quantify and genetically characterise persistent HIV. Retrovirology. 2018;15(1):3. doi: 10.1186/s12977-017-0386-x. PubMed PMID: 29316955; PMCID: PMC5761141. 29316955 PMC5761141

[49] Lian X, Seiger KW, Parsons EM, Gao C, Sun W, Gladkov GT, Roseto IC, Einkauf KB, Osborn MR, Chevalier JM, Jiang C, Blackmer J, Carrington M, Rosenberg ES, Lederman MM, McMahon DK, Bosch RJ, Jacobson JM, Gandhi RT, Peluso MJ, Chun TW, Deeks SG, Yu XG, Lichterfeld M. Progressive transformation of the HIV-1 reservoir cell profile over two decades of antiviral therapy. Cell Host Microbe. 2023;31(1):83-96.e5. doi: 10.1016/j.chom.2022.12.002. PubMed PMID: 36596305; PMCID: PMC9839361. 36596305 PMC9839361

[50] Jiang C, Lian X, Gao C, Sun X, Einkauf KB, Chevalier JM, Chen SMY, Hua S, Rhee B, Chang K, Blackmer JE, Osborn M, Peluso MJ, Hoh R, Somsouk M, Milush J, Bertagnolli LN, Sweet SE, Varriale JA, …, Yu XG. Distinct viral reservoirs in individuals with spontaneous control of HIV-1. Nature. 2020;585(7824):261-7. doi: 10.1038/s41586-020-2651-8. PubMed PMID: 32848246. 32848246 PMC7837306

[51] Einkauf KB, Osborn MR, Gao C, Sun W, Sun X, Lian X, Parsons EM, Gladkov GT, Seiger KW, Blackmer JE, Jiang C, Yukl SA, Rosenberg ES, Yu XG, Lichterfeld M. Parallel analysis of transcription, integration, and sequence of single HIV-1 proviruses. Cell. 2022;185(2):266-82.e15. doi: 10.1016/j.cell.2021.12.011. 35026153 PMC8809251

[52] Simonetti FR, White JA, Tumiotto C, Ritter KD, Cai M, Gandhi RT, Deeks SG, Howell BJ, Montaner LJ, Blankson JN, Martin A, Laird GM, Siliciano RF, Mellors JW, Siliciano JD. Intact proviral DNA assay analysis of large cohorts of people with HIV provides a benchmark for the frequency and composition of persistent proviral DNA. Proc Natl Acad Sci U S A. 2020;117(31):18692-700. doi: 10.1073/pnas.2006816117. 32690683 PMC7414172

[53] Buchholtz N, Nühn MM, de Jong TCM, Stienstra TAT, Reddy K, Ndung’u T, Ndhlovu ZM, Fisher K, Palmer S, Wensing AMJ, Symons J, Nijhuis M. Development of a highly sensitive and specific intact proviral DNA assay for HIV-1 subtype B and C. Virol J. 2024;21(1):36. doi: 10.1186/s12985-024-02300-6. PubMed PMID: 38297379; PMCID: PMC10832250. 38297379 PMC10832250

[54] Jiang S, Chan CN, Rovira-Clavé X, Chen H, Bai Y, Zhu B, McCaffrey E, Greenwald NF, Liu C, Barlow GL, Weirather JL, Oliveria JP, Nakayama T, Lee IT, Matter MS, Carlisle AE, Philips D, Vazquez G, Mukherjee N, Busman-Sahay K, Nekorchuk M, Terry M, Younger S, Bosse M, Demeter J, Rodig SJ, Tzankov A, Goltsev Y, McIlwain DR, Angelo M, Estes JD, Nolan GP. Combined protein and nucleic acid imaging reveals virus-dependent B cell and macrophage immunosuppression of tissue microenvironments. Immunity. 2022;55(6):1118-34.e8. doi: 10.1016/j.immuni.2022.03.020. PMID: 35447093; PMCID: PMC9220319. 35447093 PMC9220319

[55] Goltsev Y, Samusik N, Kennedy-Darling J, Bhate S, Hale M, Vazquez G, Black S, Nolan GP. Deep Profiling of Mouse Splenic Architecture with CODEX Multiplexed Imaging. Cell. 2018;174(4):968-81.e15. doi: 10.1016/j.cell.2018.07.010. PubMed PMID: 30078711; PMCID: PMC6086938. 30078711 PMC6086938

[56] Shaban M, Chang Y, Qiu H, Yeo YY, Song AH, Jaume G, Wang Y, Weishaupt LL, Ding T, Vaidya A. A Foundation Model for Spatial Proteomics. arXiv preprint arXiv:250603373. 2025.

[57] McCune JM, Turner EH, Jiang A, Doehle BP. Bringing Gene Therapies for HIV Disease to Resource-Limited Parts of the World. Hum Gene Ther. 2021;32(1-2):21-30. doi: 10.1089/hum.2020.252. PubMed PMID: 32998595; PMCID: PMC10112459. 32998595 PMC10112459

[58] Beacroft L, Hallett TB. The potential impact of a “curative intervention” for HIV: a modelling study. Glob Health Res Policy. 2019;4:2. doi: 10.1186/s41256-019-0107-1. PubMed PMID: 31223659; PMCID: PMC6567561. PMC6567561 31223659

[59] Griffith SA, McCoy LE. To bnAb or Not to bnAb: Defining Broadly Neutralising Antibodies Against HIV-1. Front Immunol. 2021 Oct 19;12:708227. doi: 10.3389/fimmu.2021.708227. PMID: 34737737; PMCID: PMC8560739. 34737737 PMC8560739

[60] Frattari GS, Caskey M, Søgaard OS. Broadly neutralizing antibodies for HIV treatment and cure approaches. Curr Opin HIV AIDS. 2023;18(4):157-63. doi: 10.1097/coh.0000000000000802. PubMed PMID: 37144579. 37144579

[61] Mouquet H, Scharf L, Euler Z, Liu Y, Eden C, Scheid JF, Halper-Stromberg A, Gnanapragasam PN, Spencer DI, Seaman MS, Schuitemaker H, Feizi T, Nussenzweig MC, Bjorkman PJ. Complex-type N-glycan recognition by potent broadly neutralizing HIV antibodies. Proc Natl Acad Sci U S A. 2012;109(47):E3268-77. doi: 10.1073/pnas.1217207109. PubMed PMID: 23115339; PMCID: PMC3511153. 23115339 PMC3511153

[62] Garces F, Sok D, Kong L, McBride R, Kim HJ, Saye-Francisco KF, Julien JP, Hua Y, Cupo A, Moore JP, Paulson JC, Ward AB, Burton DR, Wilson IA. Structural evolution of glycan recognition by a family of potent HIV antibodies. Cell. 2014;159(1):69-79. doi: 10.1016/j.cell.2014.09.009. PubMed PMID: 25259921; PMCID: PMC4278586. 25259921 PMC4278586

[63] Yoon H, Macke J, West AP, Jr., Foley B, Bjorkman PJ, Korber B, Yusim K. CATNAP: a tool to compile, analyze and tally neutralizing antibody panels. Nucleic Acids Res. 2015;43(W1):W213-9. doi: 10.1093/nar/gkv404. PubMed PMID: 26044712; PMCID: PMC4489231. 26044712 PMC4489231

[64] Caskey M, Schoofs T, Gruell H, Settler A, Karagounis T, Kreider EF, Murrell B, Pfeifer N, Nogueira L, Oliveira TY, Learn GH, Cohen YZ, Lehmann C, Gillor D, Shimeliovich I, Unson-O’Brien C, Weiland D, Robles A, Kümmerle T, Wyen C, Levin R, Witmer-Pack M, Eren K, Ignacio C, Kiss S, West AP Jr, Mouquet H, Zingman BS, Gulick RM, Keler T, Bjorkman PJ, Seaman MS, Hahn BH, Fätkenheuer G, Schlesinger SJ, Nussenzweig MC, Klein F. Antibody 10-1074 suppresses viremia in HIV-1-infected individuals. Nat Med. 2017;23(2):185-91. doi: 10.1038/nm.4268. PubMed PMID: 28092665; PMCID: PMC5467219. 28092665 PMC5467219

[65] Scheid JF, Mouquet H, Ueberheide B, Diskin R, Klein F, Oliveira TY, Pietzsch J, Fenyo D, Abadir A, Velinzon K, Hurley A, Myung S, Boulad F, Poignard P, Burton DR, Pereyra F, Ho DD, Walker BD, Seaman MS, Bjorkman PJ, Chait BT, Nussenzweig MC. Sequence and structural convergence of broad and potent HIV antibodies that mimic CD4 binding. Science. 2011;333(6049):1633-7. doi: 10.1126/science.1207227. PubMed PMID: 21764753; PMCID: PMC3351836. 21764753 PMC3351836

[66] Lu CL, Murakowski DK, Bournazos S, Schoofs T, Sarkar D, Halper-Stromberg A, Horwitz JA, Nogueira L, Golijanin J, Gazumyan A, Ravetch JV, Caskey M, Chakraborty AK, Nussenzweig MC. Enhanced clearance of HIV-1-infected cells by broadly neutralizing antibodies against HIV-1 in vivo. Science. 2016;352(6288):1001-4. doi: 10.1126/science.aaf1279. PubMed PMID: 27199430; PMCID: PMC5126967. 27199430 PMC5126967

[67] Schoofs T, Klein F, Braunschweig M, Kreider EF, Feldmann A, Nogueira L, Oliveira T, Lorenzi JC, Parrish EH, Learn GH, West AP, Jr., Bjorkman PJ, Schlesinger SJ, Seaman MS, Czartoski J, McElrath MJ, Pfeifer N, Hahn BH, Caskey M, Nussenzweig MC. HIV-1 therapy with monoclonal antibody 3BNC117 elicits host immune responses against HIV-1. Science. 2016;352(6288):997-1001. doi: 10.1126/science.aaf0972. PubMed PMID: 27199429; PMCID: PMC5151174. 27199429 PMC5151174

[68] Caskey M, Klein F, Lorenzi JC, Seaman MS, West AP Jr, Buckley N, Kremer G, Nogueira L, Braunschweig M, Scheid JF, Horwitz JA, Shimeliovich I, Ben-Avraham S, Witmer-Pack M, Platten M, Lehmann C, Burke LA, Hawthorne T, Gorelick RJ, Walker BD, Keler T, Gulick RM, Fätkenheuer G, Schlesinger SJ, Nussenzweig MC. Viraemia suppressed in HIV-1-infected humans by broadly neutralizing antibody 3BNC117. Nature. 2015;522(7557):487-91. doi: 10.1038/nature14411. PubMed PMID: 25855300; PMCID: PMC4890714. 25855300 PMC4890714

[69] Scheid JF, Horwitz JA, Bar-On Y, Kreider EF, Lu CL, Lorenzi JC, Feldmann A, Braunschweig M, Nogueira L, Oliveira T, Shimeliovich I, Patel R, Burke L, Cohen YZ, Hadrigan S, Settler A, Witmer-Pack M, West AP Jr, Juelg B, Keler T, Hawthorne T, Zingman B, Gulick RM, Pfeifer N, Learn GH, Seaman MS, Bjorkman PJ, Klein F, Schlesinger SJ, Walker BD, Hahn BH, Nussenzweig MC, Caskey M. HIV-1 antibody 3BNC117 suppresses viral rebound in humans during treatment interruption. Nature. 2016;535(7613):556-60. doi: 10.1038/nature18929. PubMed PMID: 27338952; PMCID: PMC5034582. 27338952 PMC5034582

[70] Lee MJ, Collins S, Babalis D, Johnson N, Falaschetti E, Prevost AT, Ashraf A, Jacob M, Cole T, Hurley L, Pace M, Ogbe A, Khan M, Zacharopoulou P, Brown H, Sutherland E, Box H, Fox J, Deeks S, Horowitz J, Nussenzweig MC, Caskey M, Frater J, Fidler S. The RIO trial: rationale, design, and the role of community involvement in a randomised placebo-controlled trial of antiretroviral therapy plus dual long-acting HIV-specific broadly neutralising antibodies (bNAbs) in participants diagnosed with recent HIV infection-study protocol for a two-stage randomised phase II trial. Trials. 2022;23(1):263. doi: 10.1186/s13063-022-06151-w. PubMed PMID: 35382844; PMCID: PMC8981886. 35382844 PMC8981886

[71] Rudicell RS, Kwon YD, Ko SY, Pegu A, Louder MK, Georgiev IS, Wu X, Zhu J, Boyington JC, Chen X, Shi W, Yang ZY, Doria-Rose NA, McKee K, O’Dell S, Schmidt SD, Chuang GY, Druz A, Soto C, Yang Y, Zhang B, Zhou T, Todd JP, Lloyd KE, Eudailey J, Roberts KE, Donald BR, Bailer RT, Ledgerwood J; NISC Comparative Sequencing Program; Mullikin JC, Shapiro L, Koup RA, Graham BS, Nason MC, Connors M, Haynes BF, Rao SS, Roederer M, Kwong PD, Mascola JR, Nabel GJ. Enhanced potency of a broadly neutralizing HIV-1 antibody in vitro improves protection against lentiviral infection in vivo. J Virol. 2014;88(21):12669-82. doi: 10.1128/JVI.02213-14. PubMed PMID: 25142607; PMCID: PMC4248941. 25142607 PMC4248941

[72] Gaudinski MR, Houser KV, Doria-Rose NA, Chen GL, Rothwell RSS, Berkowitz N, Costner P, Holman LA, Gordon IJ, Hendel CS, Kaltovich F, Conan-Cibotti M, Gomez Lorenzo M, Carter C, Sitar S, Carlton K, Gall J, Laurencot C, Lin BC, Bailer RT, McDermott AB, Ko SY, Pegu A, Kwon YD, Kwong PD, Namboodiri AM, Pandey JP, Schwartz R, Arnold F, Hu Z, Zhang L, Huang Y, Koup RA, Capparelli EV, Graham BS, Mascola JR, Ledgerwood JE; VRC 605 study team. Safety and pharmacokinetics of broadly neutralising human monoclonal antibody VRC07-523LS in healthy adults: a phase 1 dose-escalation clinical trial. Lancet HIV. 2019;6(10):e667-e79. doi: 10.1016/S2352-3018(19)30181-X. PubMed PMID: 31473167; PMCID: PMC11100866. 31473167 PMC11100866

[73] Mahomed S, Garrett N, Capparelli EV, Osman F, Harkoo I, Yende-Zuma N, Gengiah TN, Archary D, Samsunder N, Baxter C, Mkhize NN, Modise T, Carlton K, McDermott A, Moore PL, Karim QA, Barouch DH, Fast PE, Mascola JR, Ledgerwood JE, Morris L, Abdool Karim SS. Safety and Pharmacokinetics of Monoclonal Antibodies VRC07-523LS and PGT121 Administered Subcutaneously for Human Immunodeficiency Virus Prevention. J Infect Dis. 2022;226(3):510-20. doi: 10.1093/infdis/jiac041. PubMed PMID: 35134995; PMCID: PMC9417124. 35134995 PMC9417124

[74] Zhang B, Gollapudi D, Gorman J, O’Dell S, Damron LF, McKee K, Asokan M, Yang ES, Pegu A, Lin BC, Chao CW, Chen X, Gama L, Ivleva VB, Law WH, Liu C, Louder MK, Schmidt SD, Shen CH, Shi W, Stein JA, Seaman MS, McDermott AB, Carlton K, Mascola JR, Kwong PD, Lei QP, Doria-Rose NA. Engineering of HIV-1 neutralizing antibody CAP256V2LS for manufacturability and improved half life. Sci Rep. 2022;12(1):17876. doi: 10.1038/s41598-022-22435-2. PubMed PMID: 36284200; PMCID: PMC9596707. 36284200 PMC9596707

[75] Zhang B, Gorman J, Kwon YD, Pegu A, Chao CW, Liu T, Asokan M, Bender MF, Bylund T, Damron L, Gollapudi D, Lei P, Li Y, Liu C, Louder MK, McKee K, Olia AS, Rawi R, Schön A, Wang S, Yang ES, Yang Y, Carlton K, Doria-Rose NA, Shapiro L, Seaman MS, Mascola JR, Kwong PD. Bispecific antibody CAP256.J3LS targets V2-apex and CD4-binding sites with high breadth and potency. MAbs. 2023;15(1):2165390. doi: 10.1080/19420862.2023.2165390. PubMed PMID: 36729903; PMCID: PMC9897750. 36729903 PMC9897750

[76] Mahomed S, Garrett N, Karim QA, Zuma NY, Capparelli E, Baxter C, Gengiah T, Archary D, Samsunder N, Doria-Rose N, Moore P, Williamson C, Barouch DH, Fast PE, Pozzetto B, Hankins C, Carlton K, Ledgerwood J, Morris L, Mascola J, Abdool Karim S. Assessing the safety and pharmacokinetics of the anti-HIV monoclonal antibody CAP256V2LS alone and in combination with VRC07-523LS and PGT121 in South African women: study protocol for the first-in-human CAPRISA 012B phase I clinical trial. BMJ Open. 2020;10(11):e042247. doi: 10.1136/bmjopen-2020-042247. PubMed PMID: 33243815; PMCID: PMC7692975. PMC7692975 33243815

[77] Mahomed S, Garrett N, Capparelli EV, Osman F, Mkhize NN, Harkoo I, Gengiah TN, Mansoor LE, Baxter C, Archary D, Yende-Zuma N, Samsunder N, Carlton K, Narpala S, McDermott AB, Doria-Rose NA, Moore PL, Morris L, Abdool Karim Q, Mascola JR, Abdool Karim SS. Safety and pharmacokinetics of escalating doses of neutralising monoclonal antibody CAP256V2LS administered with and without VRC07-523LS in HIV-negative women in South Africa (CAPRISA 012B): a phase 1, dose-escalation, randomised controlled trial. Lancet HIV. 2023;10(4):e230-e43. doi: 10.1016/S2352-3018(23)00003-6. PubMed PMID: 37001964. 37001964

[78] Mahomed S, Garrett N, Potloane D, Sikazwe IT, Capparelli E, Harkoo I, Gengiah TN, Zuma NY, Osman F, Mansoor L, Archary D, Myeni N, Radebe P, Samsunder N, Doria-Rose N, Carlton K, Gama L, Koup RA, Narpala S, Serebryannyy L, Moore P, Williamson C, Pozzetto B, Hankins C, Morris L, Karim QA, Abdool Karim S. Extended safety and tolerability of subcutaneous CAP256V2LS and VRC07-523LS in HIV-negative women: study protocol for the randomised, placebo-controlled double-blinded, phase 2 CAPRISA 012C trial. BMJ Open. 2023;13(8):e076843. doi: 10.1136/bmjopen-2023-076843. PubMed PMID: 37640457; PMCID: PMC10462944. PMC10462944 37640457

[79] Tsai A, Irrinki A, Kaur J, Cihlar T, Kukolj G, Sloan DD, Murry JP. Toll-Like Receptor 7 Agonist GS-9620 Induces HIV Expression and HIV-Specific Immunity in Cells from HIV-Infected Individuals on Suppressive Antiretroviral Therapy. J Virol. 2017;91(8). doi: 10.1128/JVI.02166-16. PubMed PMID: 28179531; PMCID: PMC5375698. PMC5375698 28179531

[80] Lim SY, Osuna CE, Hraber PT, Hesselgesser J, Gerold JM, Barnes TL, Sanisetty S, Seaman MS, Lewis MG, Geleziunas R, Miller MD, Cihlar T, Lee WA, Hill AL, Whitney JB. TLR7 agonists induce transient viremia and reduce the viral reservoir in SIV-infected rhesus macaques on antiretroviral therapy. Sci Transl Med. 2018;10(439). doi: 10.1126/scitranslmed.aao4521. PubMed PMID: 29720451; PMCID: PMC5973480. PMC5973480 29720451

[81] SenGupta D, Brinson C, DeJesus E, Mills A, Shalit P, Guo S, Cai Y, Wallin JJ, Zhang L, Humeniuk R, Begley R, Geleziunas R, Mellors J, Wrin T, Jones N, Milush J, Ferre AL, Shacklett BL, Laird GM, Moldt B, Vendrame E, Brainard DM, Ramgopal M, Deeks SG. The TLR7 agonist vesatolimod induced a modest delay in viral rebound in HIV controllers after cessation of antiretroviral therapy. Sci Transl Med. 2021;13(599). doi: 10.1126/scitranslmed.abg3071. PubMed PMID: 34162752. 34162752

[82] Riddler SA, Para M, Benson CA, Mills A, Ramgopal M, DeJesus E, Brinson C, Cyktor J, Jacobs J, Koontz D, Mellors JW, Laird GM, Wrin T, Patel H, Guo S, Wallin J, Boice J, Zhang L, Humeniuk R, Begley R, German P, Graham H, Geleziunas R, Brainard DM, SenGupta D. Vesatolimod, a Toll-like Receptor 7 Agonist, Induces Immune Activation in Virally Suppressed Adults Living With Human Immunodeficiency Virus-1. Clin Infect Dis. 2021;72(11):e815-e24. doi: 10.1093/cid/ciaa1534. PubMed PMID: 33043969. 33043969

[83] Ndung’u T, Dong KL, Kwon DS, Walker BD. A FRESH approach: Combining basic science and social good. Sci Immunol. 2018;3(27). doi: 10.1126/sciimmunol.aau2798. PubMed PMID: 30217812; PMCID: PMC7593829. PMC7593829 30217812

[84] Ruiz L, Martinez-Picado J, Romeu J, Paredes R, Zayat MK, Marfil S, Negredo E, Sirera G, Tural C, Clotet B. Structured treatment interruption in chronically HIV-1 infected patients after long-term viral suppression. AIDS. 2000;14(4):397-403. doi: 10.1097/00002030-200003100-00013. PubMed PMID: 10770542. 10770542

[85] Davey RT, Jr., Bhat N, Yoder C, Chun TW, Metcalf JA, Dewar R, Natarajan V, Lempicki RA, Adelsberger JW, Miller KD, Kovacs JA, Polis MA, Walker RE, Falloon J, Masur H, Gee D, Baseler M, Dimitrov DS, Fauci AS, Lane HC. HIV-1 and T cell dynamics after interruption of highly active antiretroviral therapy (HAART) in patients with a history of sustained viral suppression. Proc Natl Acad Sci U S A. 1999;96(26):15109-14. doi: 10.1073/pnas.96.26.15109. PubMed PMID: 10611346; PMCID: PMC24781. 10611346 PMC24781

[86] Feher C, Leal L, Plana M, Climent N, Crespo Guardo A, Martinez E, Castro P, Diaz-Brito V, Mothe B, Lopez Bernaldo De Quiros JC, Gatell JM, Aloy P, Garcia F. Virological Outcome Measures During Analytical Treatment Interruptions in Chronic HIV-1-Infected Patients. Open Forum Infect Dis. 2019;6(12):ofz485. doi: 10.1093/ofid/ofz485. PubMed PMID: 32128329; PMCID: PMC7047957. 32128329 PMC7047957

[87] Deeks SG, Overbaugh J, Phillips A, Buchbinder S. HIV infection. Nature Reviews Disease Primers. 2015;1(1):15035. doi: 10.1038/nrdp.2015.35. 27188527

[88] Crowell TA, Colby DJ, Pinyakorn S, Sacdalan C, Pagliuzza A, Intasan J, Benjapornpong K, Tangnaree K, Chomchey N, Kroon E, de Souza MS, Tovanabutra S, Rolland M, Eller MA, Paquin-Proulx D, Bolton DL, Tokarev A, Thomas R, Takata H, Trautmann L, Krebs SJ, Modjarrad K, McDermott AB, Bailer RT, Doria-Rose N, Patel B, Gorelick RJ, Fullmer BA, Schuetz A, Grandin PV, O’Connell RJ, Ledgerwood JE, Graham BS, Tressler R, Mascola JR, Chomont N, Michael NL, Robb ML, Phanuphak N, Ananworanich J; RV397 Study Group. Safety and efficacy of VRC01 broadly neutralising antibodies in adults with acutely treated HIV (RV397): a phase 2, randomised, double-blind, placebo-controlled trial. Lancet HIV. 2019;6(5):e297-e306. doi: 10.1016/S2352-3018(19)30053-0. PubMed PMID: 31000477; PMCID: PMC6693657. 31000477 PMC6693657

[89] Bar KJ, Sneller MC, Harrison LJ, Justement JS, Overton ET, Petrone ME, Salantes DB, Seamon CA, Scheinfeld B, Kwan RW, Learn GH, Proschan MA, Kreider EF, Blazkova J, Bardsley M, Refsland EW, Messer M, Clarridge KE, Tustin NB, Madden PJ, Oden K, O’Dell SJ, Jarocki B, Shiakolas AR, Tressler RL, Doria-Rose NA, Bailer RT, Ledgerwood JE, Capparelli EV, Lynch RM, Graham BS, Moir S, Koup RA, Mascola JR, Hoxie JA, Fauci AS, Tebas P, Chun TW. Effect of HIV Antibody VRC01 on Viral Rebound after Treatment Interruption. N Engl J Med. 2016;375(21):2037-50. doi: 10.1056/NEJMoa1608243. PubMed PMID: 27959728; PMCID: PMC5292134. 27959728 PMC5292134

[90] Mendoza P, Gruell H, Nogueira L, Pai JA, Butler AL, Millard K, Lehmann C, Suárez I, Oliveira TY, Lorenzi JCC, Cohen YZ, Wyen C, Kümmerle T, Karagounis T, Lu CL, Handl L, Unson-O’Brien C, Patel R, Ruping C, Schlotz M, Witmer-Pack M, Shimeliovich I, Kremer G, Thomas E, Seaton KE, Horowitz J, West AP Jr, Bjorkman PJ, Tomaras GD, Gulick RM, Pfeifer N, Fätkenheuer G, Seaman MS, Klein F, Caskey M, Nussenzweig MC. Combination therapy with anti-HIV-1 antibodies maintains viral suppression. Nature. 2018;561(7724):479-84. doi: 10.1038/s41586-018-0531-2. PubMed PMID: 30258136; PMCID: PMC6166473. 30258136 PMC6166473

[91] Nishimura Y, Gautam R, Chun TW, Sadjadpour R, Foulds KE, Shingai M, Klein F, Gazumyan A, Golijanin J, Donaldson M, Donau OK, Plishka RJ, Buckler-White A, Seaman MS, Lifson JD, Koup RA, Fauci AS, Nussenzweig MC, Martin MA. Early antibody therapy can induce long-lasting immunity to SHIV. Nature. 2017;543(7646):559-63. doi: 10.1038/nature21435. PubMed PMID: 28289286; PMCID: PMC5458531. 28289286 PMC5458531

[92] Kelsoe G, Haynes BF. What Are the Primary Limitations in B-Cell Affinity Maturation, and How Much Affinity Maturation Can We Drive with Vaccination? Breaking through Immunity’s Glass Ceiling. Cold Spring Harb Perspect Biol. 2018;10(5). doi: 10.1101/cshperspect.a029397. PubMed PMID: 28630077; PMCID: PMC5736460. PMC5736460 28630077

[93] Nahmad AD, Lazzarotto CR, Zelikson N, Kustin T, Tenuta M, Huang D, Reuveni I, Nataf D, Raviv Y, Horovitz-Fried M, Dotan I, Carmi Y, Rosin-Arbesfeld R, Nemazee D, Voss JE, Stern A, Tsai SQ, Barzel A. In vivo engineered B cells secrete high titers of broadly neutralizing anti-HIV antibodies in mice. Nat Biotechnol. 2022;40(8):1241-9. doi: 10.1038/s41587-022-01328-9. PubMed PMID: 35681059; PMCID: PMC7613293. 35681059 PMC7613293

[94] Wu X, Yang ZY, Li Y, Hogerkorp CM, Schief WR, Seaman MS, Zhou T, Schmidt SD, Wu L, Xu L, Longo NS, McKee K, O’Dell S, Louder MK, Wycuff DL, Feng Y, Nason M, Doria-Rose N, Connors M, Kwong PD, Roederer M, Wyatt RT, Nabel GJ, Mascola JR. Rational design of envelope identifies broadly neutralizing human monoclonal antibodies to HIV-1. Science. 2010;329(5993):856-61. doi: 10.1126/science.1187659. PubMed PMID: 20616233; PMCID: PMC2965066. 20616233 PMC2965066

[95] Zhou T, Georgiev I, Wu X, Yang ZY, Dai K, Finzi A, Kwon YD, Scheid JF, Shi W, Xu L, Yang Y, Zhu J, Nussenzweig MC, Sodroski J, Shapiro L, Nabel GJ, Mascola JR, Kwong PD. Structural basis for broad and potent neutralization of HIV-1 by antibody VRC01. Science. 2010;329(5993):811-7. doi: 10.1126/science.1192819. PubMed PMID: 20616231; PMCID: PMC2981354. 20616231 PMC2981354

[96] Li Y, Migueles SA, Welcher B, Svehla K, Phogat A, Louder MK, Wu X, Shaw GM, Connors M, Wyatt RT, Mascola JR. Broad HIV-1 neutralization mediated by CD4-binding site antibodies. Nat Med. 2007;13(9):1032-4. doi: 10.1038/nm1624. PubMed PMID: 17721546; PMCID: PMC2584972. 17721546 PMC2584972

[97] Teasdale CA, Zimba R, Abrams EJ, Sachathep K, Ndagije F, Nuwagaba-Biribonwoha H, Musuka G, Mugurungi O, Maile L, Mahy M, Low A. Estimates of the prevalence of undiagnosed HIV among children living with HIV in Eswatini, Lesotho, Malawi, Namibia, Tanzania, Zambia, and Zimbabwe from 2015 to 2017: an analysis of data from the cross-sectional Population-based HIV Impact Assessment surveys. Lancet HIV. 2022;9(2):e91-e101. doi: 10.1016/S2352-3018(21)00291-5. PubMed PMID: 35120641; PMCID: PMC10350876. 35120641 PMC10350876

[98] Burrage A, Patel M, Mirkovic K, Dziuban E, Teferi W, Broyles L, Rivadeneira E. Trends in Antiretroviral Therapy Eligibility and Coverage Among Children Aged <15 Years with HIV Infection - 20 PEPFAR-Supported Sub-Saharan African Countries, 2012-2016. MMWR Morb Mortal Wkly Rep. 2018;67(19):552-5. doi: 10.15585/mmwr.mm6719a4. PubMed PMID: 29771871; PMCID: PMC6048945. 29771871 PMC6048945

[99] Johnson PR, Schnepp BC, Zhang J, Connell MJ, Greene SM, Yuste E, Desrosiers RC, Clark KR. Vector-mediated gene transfer engenders long-lived neutralizing activity and protection against SIV infection in monkeys. Nat Med. 2009;15(8):901-6. doi: 10.1038/nm.1967. PubMed PMID: 19448633; PMCID: PMC2723177. 19448633 PMC2723177

[100] Welles HC, Jennewein MF, Mason RD, Narpala S, Wang L, Cheng C, Zhang Y, Todd JP, Lifson JD, Balazs AB, Alter G, McDermott AB, Mascola JR, Roederer M. Vectored delivery of anti-SIV envelope targeting mAb via AAV8 protects rhesus macaques from repeated limiting dose intrarectal swarm SIVsmE660 challenge. PLoS Pathog. 2018;14(12):e1007395. doi: 10.1371/journal.ppat.1007395. PubMed PMID: 30517201; PMCID: PMC6296672. 30517201 PMC6296672

[101] Martinez-Navio JM, Fuchs SP, Pantry SN, Lauer WA, Duggan NN, Keele BF, Rakasz EG, Gao G, Lifson JD, Desrosiers RC. Adeno-Associated Virus Delivery of Anti-HIV Monoclonal Antibodies Can Drive Long-Term Virologic Suppression. Immunity. 2019;50(3):567-75 e5. doi: 10.1016/j.immuni.2019.02.005. PubMed PMID: 30850342; PMCID: PMC6457122. 30850342 PMC6457122

[102] Hahn PA, Martins MA. Adeno-associated virus-vectored delivery of HIV biologics: the promise of a “single-shot” functional cure for HIV infection. J Virus Erad. 2023;9(1):100316. doi: 10.1016/j.jve.2023.100316. PubMed PMID: 36915910; PMCID: PMC10005911. 36915910 PMC10005911

[103] Fetzer I, Gardner MR, Davis-Gardner ME, Prasad NR, Alfant B, Weber JA, Farzan M. eCD4-Ig Variants That More Potently Neutralize HIV-1. J Virol. 2018;92(12). doi: 10.1128/JVI.02011-17. PubMed PMID: 29593050; PMCID: PMC5974481. PMC5974481 29593050

[104] Collins DR, Gaiha GD, Walker BD. CD8(+) T cells in HIV control, cure and prevention. Nat Rev Immunol. 2020;20(8):471-82. doi: 10.1038/s41577-020-0274-9. PubMed PMID: 32051540; PMCID: PMC7222980. 32051540 PMC7222980

[105] Chea LS, Amara RR. Immunogenicity and efficacy of DNA/MVA HIV vaccines in rhesus macaque models. Expert Rev Vaccines. 2017;16(10):973-85. doi: 10.1080/14760584.2017.1371594. PubMed PMID: 28838267; PMCID: PMC6120759. 28838267 PMC6120759

[106] Rutishauser RL, Deguit CDT, Hiatt J, Blaeschke F, Roth TL, Wang L, Raymond KA, Starke CE, Mudd JC, Chen W, Smullin C, Matus-Nicodemos R, Hoh R, Krone M, Hecht FM, Pilcher CD, Martin JN, Koup RA, Douek DC, Brenchley JM, Sékaly RP, Pillai SK, Marson A, Deeks SG, McCune JM, Hunt PW. TCF-1 regulates HIV-specific CD8+ T cell expansion capacity. JCI Insight. 2021;6(3). doi: 10.1172/jci.insight.136648. PubMed PMID: 33351785; PMCID: PMC7934879. PMC7934879 33351785

[107] Medhasi S, Chantratita N. Human Leukocyte Antigen (HLA) System: Genetics and Association with Bacterial and Viral Infections. J Immunol Res. 2022;2022:9710376. doi: 10.1155/2022/9710376. PubMed PMID: 35664353; PMCID: PMC9162874. 35664353 PMC9162874

[108] Violari A, Cotton MF, Kuhn L, Schramm DB, Paximadis M, Loubser S, Shalekoff S, Da Costa Dias B, Otwombe K, Liberty A, McIntyre J, Babiker A, Gibb D, Tiemessen CT. A child with perinatal HIV infection and long-term sustained virological control following antiretroviral treatment cessation. Nat Commun. 2019;10(1):412. doi: 10.1038/s41467-019-08311-0. PubMed PMID: 30679439; PMCID: 6345921. 30679439 PMC6345921

[109] Campos-Gonzalez G, Martinez-Picado J, Velasco-Hernandez T, Salgado M. Opportunities for CAR-T Cell Immunotherapy in HIV Cure. Viruses. 2023;15(3). doi: 10.3390/v15030789. PubMed PMID: 36992496; PMCID: PMC10057306. PMC10057306 36992496

[110] Liu B, Zhang W, Xia B, Jing S, Du Y, Zou F, Li R, Lu L, Chen S, Li Y, Hu Q, Lin Y, Zhang Y, He Z, Zhang X, Chen X, Peng T, Tang X, Cai W, Pan T, Li L, Zhang H. Broadly neutralizing antibody-derived CAR T cells reduce viral reservoir in individuals infected with HIV-1. J Clin Invest. 2021;131(19). doi: 10.1172/JCI150211. PubMed PMID: 34375315; PMCID: PMC8483761. PMC8483761 34375315

[111] Mao Y, Liao Q, Zhu Y, Bi M, Zou J, Zheng N, Zhu L, Zhao C, Liu Q, Liu L, Chen J, Gu L, Liu Z, Pan X, Xue Y, Feng M, Ying T, Zhou P, Wu Z, Xiao J, Zhang R, Leng J, Sun Y, Zhang X, Xu J. Efficacy and safety of novel multifunctional M10 CAR-T cells in HIV-1-infected patients: a phase I, multicenter, single-arm, open-label study. Cell Discov. 2024;10(1):49. doi: 10.1038/s41421-024-00658-z. PubMed PMID: 38740803; PMCID: PMC11091177. 38740803 PMC11091177

[112] Herzig E, Kim KC, Packard TA, Vardi N, Schwarzer R, Gramatica A, Deeks SG, Williams SR, Landgraf K, Killeen N, Martin DW, Weinberger LS, Greene WC. Attacking Latent HIV with convertibleCAR-T Cells, a Highly Adaptable Killing Platform. Cell. 2019;179(4):880-94 e10. doi: 10.1016/j.cell.2019.10.002. PubMed PMID: 31668804; PMCID: PMC6922308. 31668804 PMC6922308

[113] Anthony-Gonda K, Bardhi A, Ray A, Flerin N, Li M, Chen W, Ochsenbauer C, Kappes JC, Krueger W, Worden A, Schneider D, Zhu Z, Orentas R, Dimitrov DS, Goldstein H, Dropulic B. Multispecific anti-HIV duoCAR-T cells display broad in vitro antiviral activity and potent in vivo elimination of HIV-infected cells in a humanized mouse model. Sci Transl Med. 2019;11(504). doi: 10.1126/scitranslmed.aav5685. PubMed PMID: 31391322; PMCID: PMC7136029. PMC7136029 31391322

[114] Anthony-Gonda K, Ray A, Su H, Wang Y, Xiong Y, Lee D, Block A, Chilunda V, Weiselberg J, Zemelko L, Wang YY, Kleinsorge-Block S, Reese JS, de Lima M, Ochsenbauer C, Kappes JC, Dimitrov DS, Orentas R, Deeks SG, Rutishauser RL, Berman JW, Goldstein H, Dropulić B. In vivo killing of primary HIV-infected cells by peripheral-injected early memory-enriched anti-HIV duoCAR T cells. JCI Insight. 2022;7(21). doi: 10.1172/jci.insight.161698. PubMed PMID: 36345941; PMCID: PMC9675454. PMC9675454 36345941

[115] Powell AB, Ren Y, Korom M, Saunders D, Hanley PJ, Goldstein H, Nixon DF, Bollard CM, Lynch RM, Jones RB, Cruz CRY. Engineered Antigen-Specific T Cells Secreting Broadly Neutralizing Antibodies: Combining Innate and Adaptive Immune Response against HIV. Mol Ther Methods Clin Dev. 2020;19:78-88. doi: 10.1016/j.omtm.2020.08.015. PubMed PMID: 33005704; PMCID: PMC7508916. 33005704 PMC7508916

[116] Chen S, van den Brink MRM. Allogeneic “Off-the-Shelf ” CAR T cells: Challenges and advances. Best Pract Res Clin Haematol. 2024;37(3):101566. doi: 10.1016/j.beha.2024.101566. PubMed PMID: 39396256. 39396256

[117] Adair JE, Androski L, Bayigga L, Bazira D, Brandon E, Dee L, Deeks S, Draz M, Dubé K, Dybul M, Gurkan U, Harlow E, Kityo C, Louella M, Malik P, Mathews V, McKemey A, Mugerwa H, Muyanja D, Olayiwola O, Orentas RJ, Popovski A, Sheehy J, Ssali F, Nsubuga MS, Tisdale JF, Verhoeyen E, Dropulić B. Towards access for all: 1st Working Group Report for the Global Gene Therapy Initiative (GGTI). Gene Ther. 2023;30(3-4):216-21. doi: 10.1038/s41434-021-00284-4. PubMed PMID: 34493840; PMCID: PMC10113145. 34493840 PMC10113145

[118] Li C, Georgakopoulou A, Newby GA, Chen PJ, Everette KA, Paschoudi K, Vlachaki E, Gil S, Anderson AK, Koob T, Huang L, Wang H, Kiem HP, Liu DR, Yannaki E, Lieber A. In vivo HSC prime editing rescues sickle cell disease in a mouse model. Blood. 2023;141(17):2085-99. doi: 10.1182/blood.2022018252. PubMed PMID: 36800642; PMCID: PMC10163316. 36800642 PMC10163316

[119] Yukselten Y, Wishah H, Li JA, Sutton RE. Targeting CCR5: A central approach to HIV treatment and cure strategies. Virology. 2025;603:110375. doi: 10.1016/j.virol.2024.110375. PubMed PMID: 39729963. 39729963

[120] Tanaka K, Kim Y, Roche M, Lewin SR. The role of latency reversal in HIV cure strategies. J Med Primatol. 2022;51(5):278-83. doi: 10.1111/jmp.12613. PubMed PMID: 36029233; PMCID: PMC9514955. 36029233 PMC9514955

[121] Clark IC, Mudvari P, Thaploo S, Smith S, Abu-Laban M, Hamouda M, Theberge M, Shah S, Ko SH, Pérez L, Bunis DG, Lee JS, Kilam D, Zakaria S, Choi S, Darko S, Henry AR, Wheeler MA, Hoh R, Butrus S, Deeks SG, Quintana FJ, Douek DC, Abate AR, Boritz EA. HIV silencing and cell survival signatures in infected T cell reservoirs. Nature. 2023;614(7947):318-25. doi: 10.1038/s41586-022-05556-6. PubMed PMID: 36599978; PMCID: PMC9908556. 36599978 PMC9908556

[122] Ren Y, Huang SH, Patel S, Alberto WDC, Magat D, Ahimovic D, Macedo AB, Durga R, Chan D, Zale E, Mota TM, Truong R, Rohwetter T, McCann CD, Kovacs CM, Benko E, Wimpelberg A, Cannon C, Hardy WD, Bosque A, Bollard CM, Jones RB. BCL-2 antagonism sensitizes cytotoxic T cell-resistant HIV reservoirs to elimination ex vivo. J Clin Invest. 2020;130(5):2542-59. doi: 10.1172/JCI132374. PubMed PMID: 32027622; PMCID: PMC7191002. 32027622 PMC7191002

[123] Arandjelovic P, Kim Y, Cooney JP, Preston SP, Doerflinger M, McMahon JH, Garner SE, Zerbato JM, Roche M, Tumpach C, Ong J, Sheerin D, Smyth GK, Anderson JL, Allison CC, Lewin SR, Pellegrini M. Venetoclax, alone and in combination with the BH3 mimetic S63845, depletes HIV-1 latently infected cells and delays rebound in humanized mice. Cell Rep Med. 2023;4(9):101178. doi: 10.1016/j.xcrm.2023.101178. PubMed PMID: 37652018; PMCID: PMC10518630. 37652018 PMC10518630

[124] Fisher BM, Cevaal PM, Roche M, Lewin SR. HIV Tat as a latency reversing agent: turning the tables on viral persistence. Front Immunol. 2025;16:1571151. doi: 10.3389/fimmu.2025.1571151. PubMed PMID: 40292298; PMCID: PMC12021871. 40292298 PMC12021871

[125] Becirovic E. Maybe you can turn me on: CRISPRa-based strategies for therapeutic applications. Cell Mol Life Sci. 2022;79(2):130. doi: 10.1007/s00018-022-04175-8. PubMed PMID: 35152318; PMCID: PMC8840918. 35152318 PMC8840918

[126] Cevaal PM, Kan S, Fisher BM, Moso MA, Tan A, Liu H, Ali A, Tanaka K, Shepherd RA, Kim Y, Ong J, Furtado DL, Holz M, Purcell DFJ, Casan JML, Payne T, Zhao W, Fareh M, McMahon JH, Deeks SG, Hoh R, Telwatte S, Pouton CW, Johnston APR, Caruso F, Symons J, Lewin SR, Roche M. Efficient mRNA delivery to resting T cells to reverse HIV latency. Nature Communications. 2025;16(1):4979. doi: 10.1038/s41467-025-60001-2. PMC12122926 40442114

[127] Suzuki Y, Ishihara H. Difference in the lipid nanoparticle technology employed in three approved siRNA (Patisiran) and mRNA (COVID-19 vaccine) drugs. Drug Metab Pharmacokinet. 2021;41:100424. doi: 10.1016/j.dmpk.2021.100424. PubMed PMID: 34757287; PMCID: PMC8502116. 34757287 PMC8502116

[128] Kilroy JM, Leal AA, Henderson AJ. Chronic HIV Transcription, Translation, and Persistent Inflammation. Viruses. 2024;16(5). doi: 10.3390/v16050751. PubMed PMID: 38793632; PMCID: PMC11125830. PMC11125830 38793632

[129] Prigann J, Tavora R, Furler O’Brien RL, Schulze-Gahmen U, Boehm D, Roan NR, Nixon DF, Ndhlovu LC, Valente S, Ott M. Silencing the transcriptionally active HIV reservoir to improve treatment outcomes. Nat Microbiol. 2024;9(10):2470-2. doi: 10.1038/s41564-024-01816-5. PubMed PMID: 39289508; PMCID: PMC11841736. 39289508 PMC11841736

[130] Mediouni S, Chinthalapudi K, Ekka MK, Usui I, Jablonski JA, Clementz MA, Mousseau G, Nowak J, Macherla VR, Beverage JN, Esquenazi E, Baran P, de Vera IMS, Kojetin D, Loret EP, Nettles K, Maiti S, Izard T, Valente ST. Didehydro-Cortistatin A Inhibits HIV-1 by Specifically Binding to the Unstructured Basic Region of Tat. mBio. 2019;10(1). doi: 10.1128/mBio.02662-18. PubMed PMID: 30723126; PMCID: PMC6368365. PMC6368365 30723126

[131] Kessing CF, Nixon CC, Li C, Tsai P, Takata H, Mousseau G, Ho PT, Honeycutt JB, Fallahi M, Trautmann L, Garcia JV, Valente ST. In Vivo Suppression of HIV Rebound by Didehydro-Cortistatin A, a “Block-and-Lock” Strategy for HIV-1 Treatment. Cell Rep. 2017;21(3):600-11. doi: 10.1016/j.celrep.2017.09.080. PubMed PMID: 29045830; PMCID: PMC5653276. 29045830 PMC5653276

[132] Passi A, Tibocha-Bonilla JD, Kumar M, Tec-Campos D, Zengler K, Zuniga C. Genome-Scale Metabolic Modeling Enables In-Depth Understanding of Big Data. Metabolites. 2021;12(1). doi: 10.3390/metabo12010014. PubMed PMID: 35050136; PMCID: PMC8778254. PMC8778254 35050136

[133] Ambikan AT, Svensson-Akusjarvi S, Krishnan S, Sperk M, Nowak P, Vesterbacka J, Sonnerborg A, Benfeitas R, Neogi U. Genome-scale metabolic models for natural and long-term drug-induced viral control in HIV infection. Life Sci Alliance. 2022;5(9). doi: 10.26508/lsa.202201405. PubMed PMID: 35537851; PMCID: PMC9095731. PMC9095731 35537851

[134] Mikaeloff F, Gelpi M, Escós A, Wang T, Gupta S, Olofsson A, Akusjärvi SS, Schuster S, Naval P, Sood V, Nikouyan N, Knudsen AD, Vestad B, Høgh J, Hov JR, Benfield T, Trøseid M, Pawar V, Rucevic M, Benfeitas R, Végvári Á, O’Mahony L, Savai R, Björkström NK, Lourda M, de Magalhães JP, Weiss S, Mardinoglu A, Varshney MK, Karlsson AC, Syed YA, Nielsen SD, Neogi U. Host Plasma Microenvironment in Immunometabolically Impaired HIV Infection Leads to Dysregulated Monocyte Function and Synaptic Transmission Ex Vivo. Adv Sci (Weinh). 2025;12(16):e2416453. doi: 10.1002/advs.202416453. PubMed PMID: 40013867; PMCID: PMC12021100. 40013867 PMC12021100

[135] Dybul M, Attoye T, Baptiste S, Cherutich P, Dabis F, Deeks SG, Dieffenbach C, Doehle B, Goodenow MM, Jiang A, Kemps D, Lewin SR, Lumpkin MM, Mathae L, McCune JM, Ndung’u T, Nsubuga M, Peay HL, Pottage J, Warren M, Sikazwe I; Sunnylands 2019 Working Group. The case for an HIV cure and how to get there. Lancet HIV. 2021;8(1):e51-e8. doi: 10.1016/S2352-3018(20)30232-0. PubMed PMID: 33271124; PMCID: PMC7773626. 33271124 PMC7773626

[136] Brink DT, Martin-Hughes R, Bowring AL, Wulan N, Burke K, Tidhar T, Dalal S, Scott N. Impact of an international HIV funding crisis on HIV infections and mortality in low-income and middle-income countries: a modelling study. Lancet HIV. 2025;12(5):e346-e54. doi: 10.1016/S2352-3018(25)00074-8. PubMed PMID: 40157378. 40157378

